# Increased Angiogenin Expression Correlates With Radiation Resistance and Predicts Poor Survival for Patients With Nasopharyngeal Carcinoma

**DOI:** 10.3389/fphar.2021.627935

**Published:** 2021-08-26

**Authors:** Shan-Shan Guo, Yu-Jing Liang, Li-Ting Liu, Qiu-Yan Chen, Yue-Feng Wen, Sai-Lan Liu, Xue-Song Sun, Qing-Nan Tang, Xiao-Yun Li, Hai-Qiang Mai, Lin-Quan Tang

**Affiliations:** ^1^ State Key Laboratory of Oncology in South China, Guangdong Key Laboratory of Nasopharyngeal Carcinoma Diagnosis and Therapy, Sun Yat-sen University Cancer Center, Collaborative Innovation Center for Cancer Medicine, Guangzhou, China; ^2^ Department of Nasopharyngeal Carcinoma, Sun Yat-sen University Cancer Center, Guangzhou, China; ^3^ Department of Radiotherapy, Affiliated Cancer Hospital and Institute of Guangzhou Medical University, Guangzhou, China

**Keywords:** angiongenin, biomarker, radio-resistance, nasopharyngeal carcinoma, prognosis

## Abstract

**Background:** Despite the development of such multiple therapeutic approaches, approximately 20% patients experience recurrence. Identification of molecular markers for stratifying the different risks of tumour recurrence and progression is considered imperative.

**Methods:** We used a RayBio Human Cytokine Antibody Array that simultaneously detected the levels of 297 proteins and profiled the conditioned medium of HONE1 cells and the radioresistant NPC cells HONE1-IR. We found Angiogenin(ANG) expression to be significantly increased in HONE1-IR and HONE1-IR cells exposed to 4-Gy X-ray radiation.

**Results:** We investigated the expression of ANG in NPC tissues and explored its prognostic significance in patients with NPC. We found that ANG expression was increased in recurrent NPC tissues. Elevated expression of ANG induced radio-resistance in NPC cells, in addition to being significantly associated with shorter PFS, OS, and LRFS in patients with NPC. Multivariate analysis results revealed that ANG was an independent prognostic factor that predicted PFS, OS, and LRFS. Furthermore, a nomogram model was generated to predict OS in terms of ANG expression.

**Conclusion:** Our results found the radioresistant function of ANG and proved the clinical prognostic significance of ANG, and the results could help predict radio-sensitivity and stratify high-risk patients or tumour recurrence.

## Background

Nasopharyngeal carcinoma (NPC) is endemic in southern China and South-East Asia (Chen et al., 2019). Radiotherapy (RT) is the primary treatment for the non-metastatic disease. For early-stage NPC, RT alone is the recommended treatment, whereas for locoregional NPC, concurrent chemoradiotherapy (CCRT) and induction chemoradiotherapy (IC) + CCRT constitute the standard treatment strategy (Lam and Chan, 2018). Despite the development of such multiple therapeutic approaches, approximately 20% patients experience recurrence (Lee et al., 2019). Although the American joint Committee on cancer (AJCC) staging system can differentiate the prognoses for patients with NPC, patients with the same tumour stage may also show heterogeneity in clinical outcomes. Therefore, identification of molecular markers for stratifying the different risks of tumour recurrence and progression is considered imperative. Development of novel biomarkers might enable better prediction of prognosis for patients with NPC and help develop potential therapeutic targets.

Angiogenin (ANG) is a secreted ribonuclease best known for its ability to promote blood vessel formation (Lyons et al., 2017). It has been reported to promote the metastasis of colorectal cancer cells (Li et al., 2019), proliferation and invasion of lung squamous carcinoma cells (Xu et al., 2016), and tumorigenesis in bladder cancer (Peres et al., 2016). Additionally, ANG can mediate tumour angiogenesis in prostate cancer and the proliferation in prostate cancer cells (Vanli and Guo-Fu, 2015). Recently, serum ANG level has been found to be a prognostic factor for numerous tumours, such as glioblastoma (Hu et al., 2019), non-Hodgkin lymphoma (Fang et al., 2011), cervical cancer (Landt et al., 2011), and laryngeal carcinoma (Marioni et al., 2010). Gino et al. investigated ANG by immunohistochemistry (IHC) in 15 Caucasian patients with NPC and found a trend towards significant inverse correlation between ANG expression and disease-free survival (Marioni et al., 2010). However, due to the very small sample size and all patients being of Caucasian origin, the results of this study would require further validation by other studies with larger sample size in prevalent districts. To date, no study has investigated the prognostic value of ANG in a prevalent district. Hence, exploring the prognostic significance of ANG for patients with NPC in a prevalent district was considered necessary.

In the present study, we used a RayBio Human Cytokine Antibody Array that simultaneously detected the levels of 297 proteins and profiled the conditioned medium of HONE1 cells and the radioresistant NPC cells HONE1-IR. We found ANG expression to be significantly increased in HONE1-IR and HONE1-IR cells exposed to 4-Gy X-ray radiation. Subsequently, we investigated the expression of ANG in NPC tissues and explored its prognostic significance in patients with NPC. Finally, we hypothesised that ANG expression could be a potential prognostic factor for patients with NPC.

## Materials and Methods

### Ethics Statement

This study was approved by the Institutional Review Board of Sun Yat-Sen University Cancer Center. All patients signed a written informed consent for the collection of tissue samples.

### Patient Recruitment and Follow-Up

The expression of ANG in tissue cells was evaluated in NPC tumour samples that were obtained before treatment from 175 histologically confirmed patients with NPC, who were prospectively enrolled between January 2010 and November 2011. Entry criteria for patients were as follows: histologically proven NPC, with stage I–IVa (according to the eighth edition of the AJCC/UICC staging system), no distant metastasis at diagnosis, no anti-cancer treatment received prior to admission, no other tumour types or serious illness, and radical intensity-modulated radiotherapy (IMRT) received during treatment. Staging examinations included the following: magnetic resonance imaging of head and neck, chest radiograph, bone scintigraphy, and ultrasonography of the abdominal region of all patients. The median age of all the included patients was 49 years, ranging from 19 to 70 years. There were 137 male and 38 female patients, with a sex ratio of 3.6:1. The characteristics of the 175 patients with NPC were shown in Table 1.

**TABLE 1:** Characteristics of the NPC patients.; Abbreviation: CCRT, concurrent chemoradiotherapy; ECOG, Eastern Cooperative Oncology Group; IC, induction chemotherapy; NPC, nasopharyngeal carcinoma; RT, radiotherapy; WHO, World Health Organization.

Characteristics	Angiogenin		*p* Value
	Low expression	High expression	
Age			0.811
≤49	46	40	
>49	46	43	
Gender			
Female	18	20	0.468
Male	74	63	
T stage			0.499
1	4	6	
2	11	15	
3	50	38	
4	27	24	
N stage			0.537
0	18	17	
1	26	29	
2	36	24	
3	12	13	
Clinical stage			0.327
1	2	2	
2	8	14	
3	41	36	
4	41	31	
WHO Type			0.06
1	0	1	
2	5	0	
3	87	80	
ECOG score			1.000
0	2	2	
1	90	81	
Smoking history			0.816
0	55	53	
1	37	30	
EBV DNA			0.811
≤4,000	46	40	
>4,000	46	43	
Treatment strategy			0.43
RT	10	12	
CCRT	35	24	
IC + CCRT	47	47	

The treatment regimens included RT alone, CCRT, and IC + CCRT. IMRT was performed for all the included patients, in accordance with the treatment policy for NPC at Sun Yat-Sen University Cancer Center (SYSUCC). The chemotherapy regimen used for IC was PF (intravenous (IV) administration of 80–100 mg/m^2^ cisplatin on day 1 and that of 800 mg/m^2^/d 5-Fu continuously over days 1–5). The chemotherapy regimen was repeated every 3 weeks for 2–3 cycles. Concurrent chemotherapy primarily included the IV administration of 80–100 mg/m^2^ cisplatin every 3 weeks.

After the completion of treatment, patients were followed up every month for the first 3 months, every 3 months over 3 years, every 6 months for the next 2 years, and annually thereafter. Median follow-up time for the patients was 83 months (range, 5–106 months).

### IHC

The antibody used in the study was anti-ANG (cat. no. 0555–5,008, AbD Serotec, MorphoSys, Oxford, United Kingdom). Tissue sections were de-paraffinised with xylene and rehydrated with ethanol. Hydrogen peroxide (3%) was used to remove any endogenous peroxidase. Tissue slices were incubated with pepsin (no. ZLI-9013, ZSGB-Bio, Beijing, China) at 37°C for 20 min for antigen retrieval. The samples were incubated with the primary antibodies (1:100 dilution) overnight at 4°C. The sections were then washed with PBS and incubated with secondary antibodies (EnVision, Dako, Carpinteria, CA, United States) for 20 min at 37 °C; 3,3′-Diaminobenzidine was used to visualise the antigens. Sections were counterstained with haematoxylin. Hydrochloric acid-alcohol was used for differentiation. The scoring system for IHC, to generate the immunoreactivity score, was used as described previously (Waisberg et al., 2014). Two independent pathologists, without prior knowledge of the clinical origin of the specimen, evaluated each specimen.

### Cell Lines and Culture Conditions

The NPC cell line, HONE1, and the radioresistant NPC cell line, HONE1-IR, were cultured in RPMI 1640 medium (Gibco) supplemented with 5% foetal bovine serum (FBS; Gibco). The cell lines were cultured in a humidified incubator containing 5% CO_2_ at 37°C. Regular morphological observations and tests for the absence of mycoplasma contamination (MycoAlert, Lonza) were conducted for the authentication of all the cell lines used in this study. The radioresistant NPC cell line HONE1-IR has been validated in our previous study (Guo et al., 2020).

### Small Interfering RNA Transfection

Transient transfection of HONE1-IR cells was performed using Lipofectamine RNAiMax (cat. no. 1795160; Invitrogen) according to the manufacturer’s instructions. HONE1-IR cells were seeded onto six-well plates at a density of 1.5 × 10^5^ cells/well 1 day prior to transfection. They were transfected with 50 pmol siRNA and subjected to clone formation at 24 h post-transfection. Scramble siRNA was purchased from Ruibo (Guangzhou, China). The siRNA sequences for *ANG* was 5′-CGT​TGT​TGT​TGC​TTG​TGA​A-3′.

### Western Blot Analysis

Cells were collected and lysed in sodium dodecyl sulphate (SDS) sample buffer (62.5 mM Tris-HCl [pH 6.8], 3% SDS, 10% glycerol, 50 mM dl-dithiothreitol, and 0.1% bromophenol blue) containing protease inhibitors (Roche, Indianapolis, IN, United States). Protein concentrations were tested using the BCA method (Pierce, Thermo Fisher Scientific, Rockford, IL, United States). Proteins (20 μg) were separated by SDS-polyacrylamide gel electrophoresis and transferred to polyvinylidene difluoride membranes. Bovine serum albumin (5%) in TBS-T (1 M Tris-HCl [pH 7.5], 0.8% NaCl, and 0.1% Tween 20) was used to block the membranes. The membranes were then incubated with primary antibodies (1:100 dilution) at 4°C overnight, followed by incubation with horseradish peroxidase-conjugated secondary antibodies (Pierce). Proteins were then visualised by enhanced chemiluminescence (Pierce). Antibodies against β-actin (cat. no. 66009-1-Ig; Proteintech) and ANG (cat. no. 0555–5,008, AbD Serotec, Kidlington, United Kingdom) were used in the experiments.

### Clonogenic Assay for Radio-Sensitivity and Irradiation

A total of 1 × 10^3^ cells were seeded onto 3-cm dishes and incubated for 10 days after exposure to various doses of irradiation. After visual verification of colony formation, cells were briefly stained with 0.1% crystal violet in 100% methanol. Colonies that consisted of 100 or more cells were counted as clonogenic survivors. The surviving fraction was calculated by dividing the number of colonies by the number of seeded cells and then multiplying it by the plating efficiency, which is defined as the (number of colonies formed/number of cells seeded) × 100%. All the experiments were repeated independently at least thrice. An X-ray irradiation instrument with 4.2 kW X-rays (RS 2000; Rad Source Technologies Inc.), available at SYSUCC, was used to irradiate the cells. The uniformity of irradiation was appreciable, and the difference was less than 5%. Dose rate for irradiating the cells was 1.26 Gy/min.

### Cell Cycle and Cell Apoptosis Analysis

Cell cycle was studied on HONE1 cells as control and knock-down the expression of ANG by siRNA interfering at 48 h post-culture, the 5 × 10^5^ cells plated in 6 wells plate were harvested, fixed in 70% ethanol over-night and stained with 500 μl of propidium iodure for 30 min at 37°C. Apoptosis analysis was performed by using FITC Annexin V Apoptosis Detection Kit with PI(Catalog: 556547, Lot: 4136994, BD Biosciences) according to the manufacturer’s instructions. Samples were analyzed by flow cytometry (FACS, AriaIII, and BD, United States) and all tests were repeated three times (in Triplicate).

### Statistical Analysis

A chi-squared analysis was used to compare the incidence rates with categorical variables. Survival rates were calculated using the Kaplan-Meier method and compared using log-rank tests. Multivariate analyses were performed using the Cox proportional hazards model. Hazard ratio point and interval (95% confidence interval) estimates were computed using the Cox proportional hazards model. ANG expression was adjusted for patient sex, age, T stage, N stage, UICC stage, and EBV DNA in the Cox proportional hazards model. Locoregional recurrence-free survival (LRFS) was defined as the time interval from the beginning of treatment to the date of first observation of local and/or regional recurrence or censored at the date of the last follow-up. Distant metastasis-free survival (DMFS) was defined as the time interval from the beginning of treatment to the date of first observation of distant lesions or censored at the date of last follow-up. Progression-free survival (PFS) was defined as the time interval from the beginning of treatment to the date of first observation of recurrence, distant metastasis, or death; alternatively, PFS was censored at the date of last follow-up. Overall survival (OS) was defined as the time interval from the beginning of treatment to the date of death or censored at the date of last follow-up.

The Statistical Package for Social Sciences version 18.0 software program (SPSS Inc., Chicago, IL, United States) was used for our analysis. A prognostic nomogram was established with significant variables for OS in the Cox regression model using the survival and rms package in R 4.0.0. The predictive accuracy and discriminative ability of this nomogram were evaluated using the C-index. All *p*-values were two sided, and p < 0.05 was considered statistically significant.

## Results

### ANG-Mediated Radio-Resistance in NPC Cells *In vitro* and its Correlation With NPC Recurrence

**FIGURE 1:**
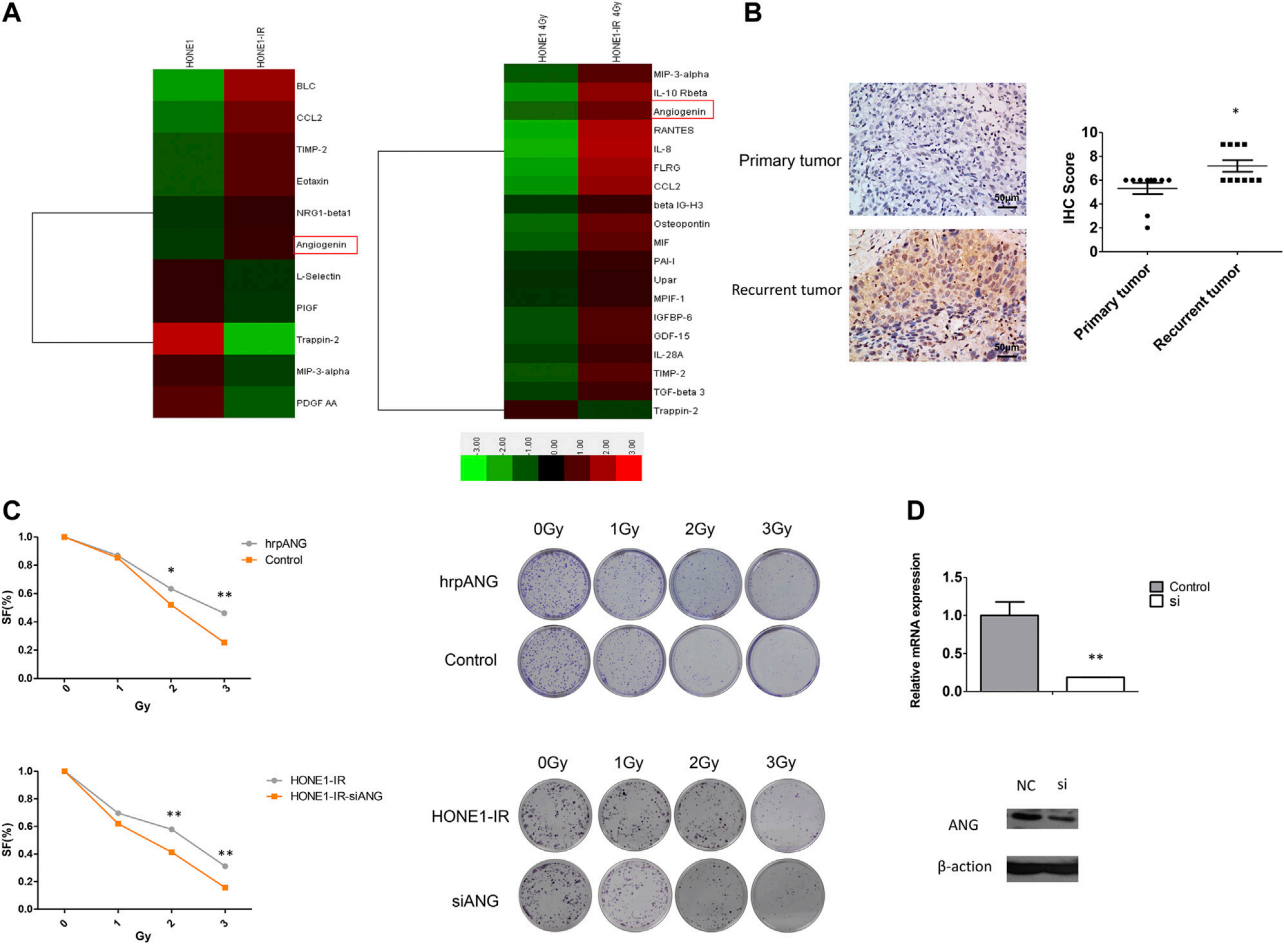
Angiogenin (ANG) expression was elevated in radioresistant NPC cells and recurrent tumour tissues. **(A)** Heat maps show the expression pattern of 11 genes in HONE1 vs. HONE1-IR cells and 19 genes in HONE1 vs. HONE1-IR cells after 4-Gy irradiation, derived from unsupervised clustering analysis. Red or green reflects high or low expression, respectively, as indicated in the scale bar (Raw Z score). **(B)** The differential IHC scores of tumour tissues, regarding ANG expression, from 10 patients with NPC before initial treatment and after tumour recurrence in the same patients. * p < 0.05 following nonparametric tests. **(C)** The human recombinant protein ANG (hrp-ANG) led to increased radio-resistance, as seen by colony formation assays in HONE1 cells (upper panel). Radiation sensitivity was tested by colony formation assays in HONE1-IR cells and those transfected with CCL2-siRNAs (lower panel). Survival curves are constructed based on means ± SEM; * p < 0.05, ** p < 0.001. **(D)** ANG mRNA expression and protein expression were significantly decreased in HONE1-IR cells transfected with ANG-siRNAs.

Previously, we had used cytokine antibody array test on radioresistant NPC cell line HONE-IR and its original cell line HONE1, as mentioned in our previous study (Guo et al., 2020), and found ANG to be significantly elevated in HONE1-IR cells, and significantly increased further by radiation (Figure 1A). Therefore, we speculated ANG to be correlated with radio-resistance and tumour recurrence in NPC. Ten pairs of tumour tissues from primary NPC and normal tissues from the same patient, presenting tumour recurrence, were tested by IHC. The IHC scores showed ANG expression in recurrent NPC tissues to be significantly higher than that in primary tumour tissues (Figure 1B).

Since ANG is a secreted protein, we used hrp-ANG (cat. no. 265-AN; R&D Systems, Minneapolis, MN, USA) to investigate whether ANG could modulate radio-resistance in NPC cells. We found hrp-ANG-treated HONE1 cells to present increased resistance to IR than control cells (Figure 1C upper panel). In addition, we used specific siRNAs to knock down ANG in HONE1-IR cells; colony formation assays indicated ANG knockdown to markedly decrease colony formation after irradiation (Figure 1C lower panel). The efficacy of siRNAs for knocking down ANG expression is shown in Figure 1D.

### ANG as an Independent Prognostic Factor for Clinical Outcomes in Patients With NPC

**FIGURE 2:**
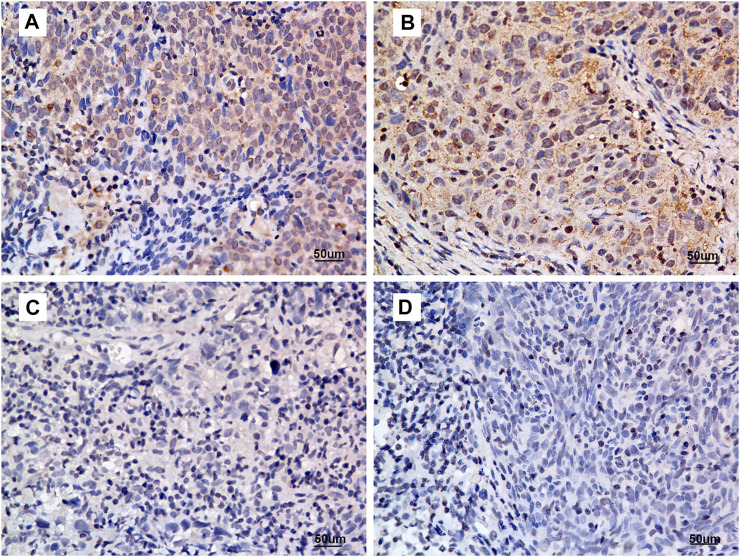
Criteria for ANG expression intensity scoring. **(A)** intensity score = 3, **(B)** intensity score = 2, **(C)** intensity score = 1, and **(D)** intensity score = 0. Representative micrographs are shown (400×). All micrographs were collected and processed under identical conditions.

The IHC scores of 175 primary tumour tissues were analysed to identify the prognostic value of ANG in NPC. Figure 2 presents the intensity score of ANG expression in NPC tissues. The baseline data for patient characteristics were well balanced (Table 1). The study included 38 women (21.7%) and 137 men (78.3%), with a median age of 49 years (range, 19–78 years). Four patients (2.3%) were classified to be in stage I, 22 (12.6%) in stage II, 77 (44.0%) in stage III, and 72 (41.1%) in stage IV. The median follow-up time was 83 months (range, 5–106 months).

After regular follow-up, a total of 44 patients presented tumour progression, 31 presented distant metastasis, eight presented locoregional recurrence, and 31 died. For all the included patients, 5-years OS was 86.5%, 5-years PFS was 77.0%, 5-years LRFS was 91.1%, and 5-years DMFS was 83.3%.

**FIGURE 3:**
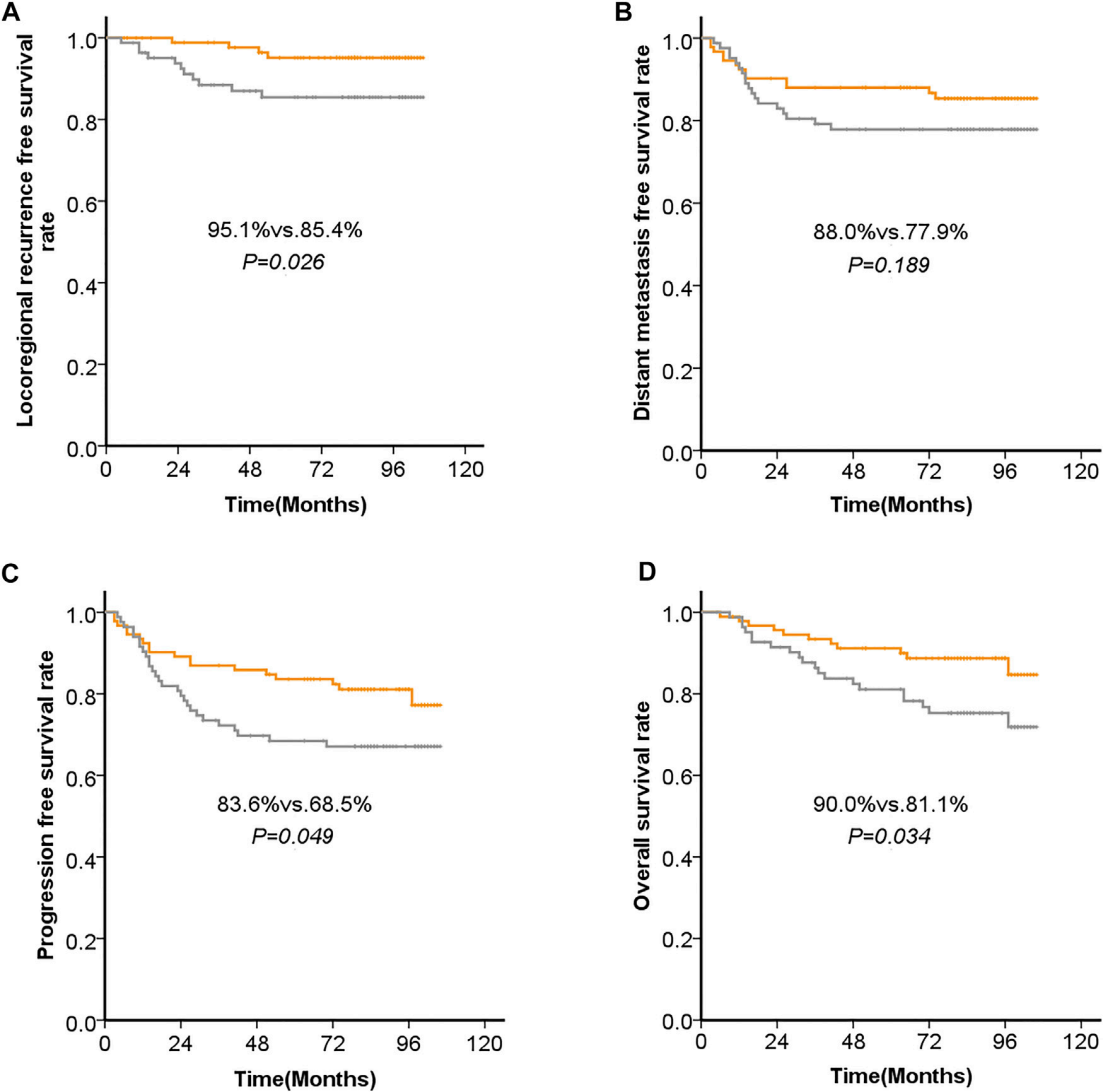
Kaplan-Meier analysis of **(A)** the 5-years locoregional recurrence-free survival (LRFS), **(B)** 5-years distant metastasis-free survival (DMFS), **(C)** progression-free survival (PFS), and **(D)** overall survival (OS), with regard to the expression levels of ANG in patients with NPC before initial treatment.

Five-year LRFS was significantly better in the low ANG-expression group than in the high ANG-expression group (95.1 vs. 85.4%, *p* = 0.026). Similarly, 5-year PFS was significantly better in the low ANG-expression group than in the high ANG-expression group (83.6 vs. 68.5%, *p* = 0.049). Five-year OS was also significantly better in the low ANG-expression group than in the high ANG-expression group (90.0 vs. 81.1%, *p* = 0.034). However, there was no significant difference between the low and high ANG-expression groups regarding 5-years DMFS. The survival curves based on Kaplan-Meier method, for patients with NPC, regarding ANG expression level, are shown in Figure 3.

In multivariate analysis, ANG expression was significantly associated with 5-years OS, PFS, and LRFS. Results from the multivariate Cox regression analysis are shown in Table 2. ANG expression was significantly associated with 5-years OS (p = 0.022), HR of 2.428 (95%CI, 1.134–5.200); PFS (*p* = 0.014), HR of 2.160 (95%CI, 1.165–4.005); and LRFS (*p* = 0.028), HR of 3.767 (95%CI, 1.153–12.309). Additionally, the results of multivariate analysis revealed that T stage, N stage, and EBV DNA were significantly associated with OS and DMFS, while T stage and N stage were significantly associated with PFS. The results of multivariate analysis of Cox proportional hazard regression were shown in Table 2.

**TABLE 2:** The multivariate analysis of Angiogenin in NPC patients.

	HR	95%CI	*p* Value
LRFS			
Age	1.420	0.487–4.139	0.521
Gender	0.526	0.155–1.780	0.302
T stage	1.507	0.502–4.524	0.465
N stage	1.204	0.642–2.256	0.563
Clinical stage	1.867	0.546–6.386	0.320
EBV DNA	1.070	0.301–3.805	0.916
Angiogenin	3.767	1.153–12.309	0.028
DMFS			
Age	1.212	0.594–2.474	0.596
Gender	1.416	0.407–4.931	0.584
T stage	2.968	1.326–6.644	0.008
N stage	2.017	1.220–3.334	0.006
Clinical stage	0.698	0.270–1.802	0.457
EBV DNA	2.998	1.251–7.184	0.014
Angiogenin	1.636	0.772–3.468	0.199
OS			
Age	1.044	0.511–2.133	0.906
Gender	1.408	0.407–4.874	0.589
T stage	4.581	1.752–11.978	0.002
N stage	1.987	1.236–3.196	0.005
Clinical stage	0.480	0.165–1.398	0.178
EBV DNA	2.793	1.243–6.278	0.013
Angiogenin	2.428	1.134–5.200	0.022
PFS			
Age	1.168	0.647–2.109	0.606
Gender	0.792	0.354–1.772	0.570
T stage	2.387	1.251–4.557	0.008
N stage	1.661	1.140–2.422	0.008
Clinical stage	1.103	0.529–2.297	0.794
EBV DNA	1.877	0.959–3.674	0.066
Angiogenin	2.160	1.165–4.005	0.014

Abbreviations: CI, confidence interval; DMFS, distant metastasis free survival; GPS, glasgow prognostic score; HR, hazard ratio; LRFS, locoregional recurrence free survival; OS, overall survival; PFS, progression free survival.

### Prognostic Nomogram for OS

**FIGURE 4:**
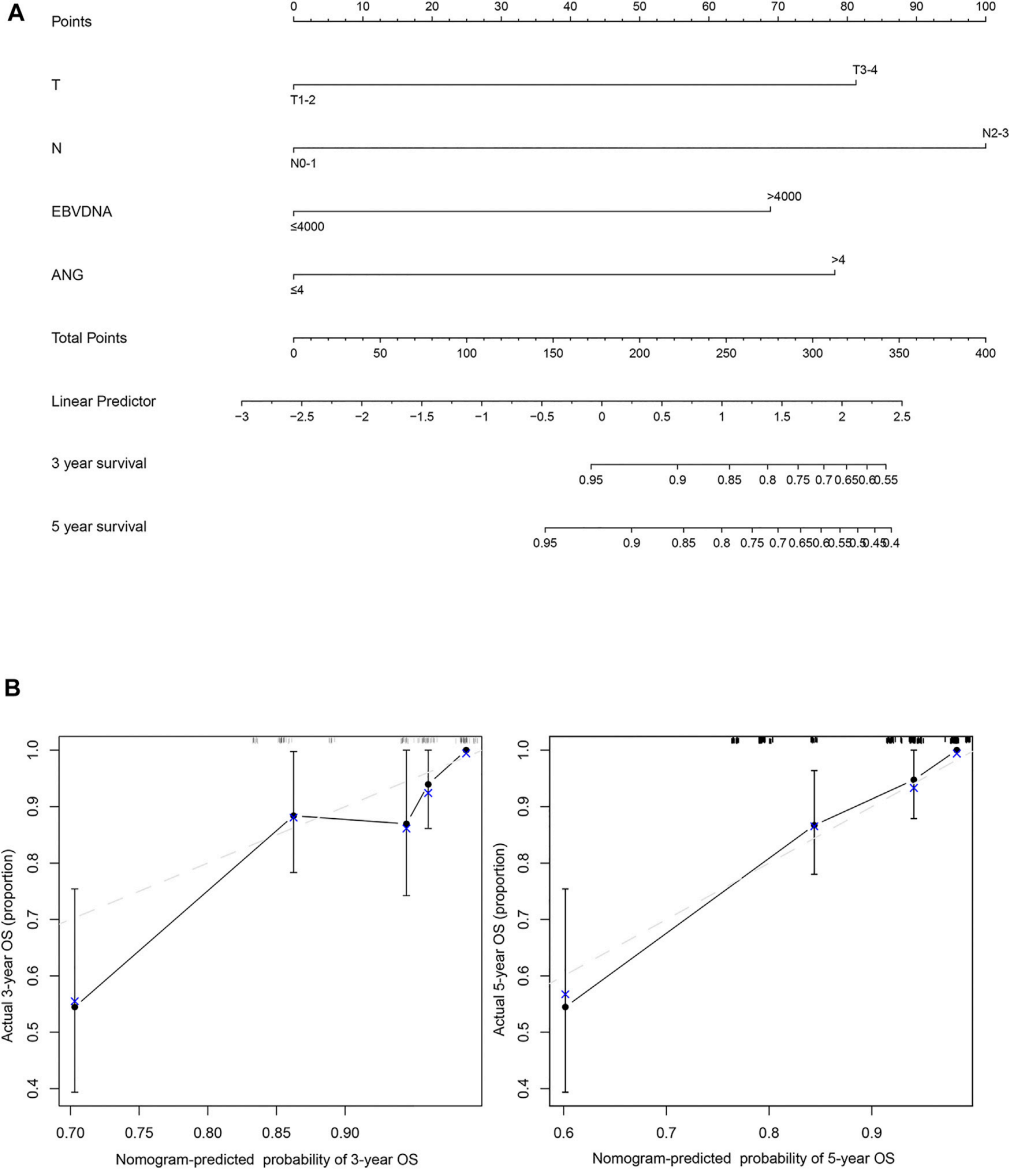
The nomogram and model calibration curve. **(A)** Nomogram including T stage, N stage, EBV DNA, and ANG expression levels in patients with nasopharyngeal carcinoma. **(B)** Model calibration curve showing the predicted and actual probabilities of ANG expression.

A prognostic nomogram for OS was established, containing significant prognostic variables such as T stage, N stage, EBV DNA, and ANG (Figure 4A). The nomogram showed T stage and N stage to contribute the most to the prognosis of OS, whereas ANG and EBV DNA were also found to play important roles. Each subtype of the aforementioned variables was assigned a grade-point score. A straight line could easily be drawn to determine the estimated proportion of OS rate at each time point by adding up the total score and positioning it on the total score scale. Harrell’s C-index of the established nomogram (to predict OS) was 0.80 (95% CI, 0.73–0.87), displaying a model with favourable discriminative capacity. Calibration curves for the 3-years and 5-years OS probabilities displayed optimal agreement between the actual observed survival rate and nomogram prediction (Figure 4B).

## Discussion

Although the local control of NPC can be increased by IMRT rather than conventional radiotherapy, approximately 20% of the patients still present locoregional recurrence following radical IMRT (Lee et al., 2019). Survival of the recurrent patients with NPC remains low, and the side effects after re-radiotherapy as a treatment for recurrent NPC are severe (e.g., bleeding) (Karam et al., 2016). Tumour recurrence has been recommended to have relationship strong association with radio-resistance (Barker et al., 2015). Any biomarker that can help identify radio-sensitivity would be useful in this context; therefore, possible candidates for predicting tumour recurrence and progression are urgently required.

In the present study, we found ANG to promote radio-resistance in NPC cells. In addition, we found ANG to be an independent prognostic factor for OS, PFS, and LRFS. To the best of our knowledge, this is the first study to identify the radioresistant function and prognostic significance of ANG in NPC. The results of our study could help stratify patients with different degrees of risk and guide clinical treatment strategies.

The possible mechanism underlying ANG-promoted radio-resistance in NPC cells is as follows: Since microvesicles derived from mesenchymal stem cells are known to promote angiogenesis, Chen et al. (2014) used an antibody array and found that Angiogenin, VEGF, IGF, Tie-2/TEK, and IL-6 which were higher in microvesicles under hypoxic conditions than under normoxic conditions, which revealed that ANG might be responsible for the hypoxia-augmented proangiogenic effects of microvesicles. In addition, Chang et al. (2017) found several angiogenic cytokines in the medium, including ANG, which promoted the formation of tubules across human umbilical cord vein endothelial cells and protected the cells against radiation-induced apoptosis *in vitro.* Yamasaki et al. (2009) showed ANG and tiRNAs to participate in a process of stress-induced translational repression. ANG may be a stress-induced factor that protects adjacent or distant cells from the deleterious effects of environmental stress. Studies have demonstrated ANG to induce cell survival, proliferation, endothelial tube formation, xenograft angiogenesis, cell migration, angiogenesis, and tumour suppressor gene expression (Miyake et al., 2015; Peres et al., 2016). Hence, ANG may protect NPC cells from the radiation-induced hypoxia-modulated environmental stress and induce cell survival, proliferation, and radio-resistance. However, the exact mechanism warrants further study. As we further did experiments of cell cycle showed that more cells stayed in S and G2/M phase after siRNA interfered ANG in HONE1 cells (Supplementary Figure S1A). So we speculated that knocked down ANG lead to more cells stayed in G2/M to be more radiosensitive, as previous studies mentioned that cells being most radiosensitive in the G (2)-M phase, less sensitive in the G (1) phase (Pawlik and Keyomarsi, 2004). In addition, knocked down the expression of ANG contributed to cell apoptosis as shown in Supplementary Figure S1B, revealed that ANG’s anti-apoptosis function, which was in accordance with previous studies (Li et al., 2014; Shu-Ping and Guo-Fu, 2015). It is possible that the function on cell cycle and apoptosis of ANG results in the radioresistant function of ANG. We also tested the cell migration after knocked down ANG expression and found that ANG promoted cell migration, which was in accordance with previous studies (Schwartz et al., 2007; Li et al., 2019) and maybe it is associated with its angiogenesis function and accelerated metastasis. Further studies are in urgently need to explain this results.

In the current study, we found the IHC scores of ANG in recurrent NPC tissues to be significantly higher than those in primary tumour tissues, and ANG expression was significantly associated with tumour recurrence in NPC. Since previous studies have indicated tumour recurrence to be related to radio-resistance (Chen et al., 2014; Yang et al., 2020), these findings collectively indicate that ANG expression may be an important biomarker of radio-resistance in patients with NPC.

Previous studies on other tumours have found ANG expression to be a prognostic factor for tumour recurrence, tumour progression, and OS. For example, a study analysed ANG expression in 108 operable laryngeal squamous cell carcinoma tissues and found ANG expression to be related to carcinoma recurrence rate and disease-free survival (DFS) (Marioni et al., 2010). Previous studies have found ANG expression to be significantly high in cases with loco-regional recurrent disease in laryngeal squamous cell carcinoma (Marioni et al., 2014; Lovato et al., 2015); ANG expression ≥ 5.0% is considered a significant, independent, negative prognostic factor in terms of DFS (Chen et al., 2014; Yang et al., 2020). Hu et al. found high ANG expression to be an independent indicator of shorter OS in proneural glioblastoma (Chen et al., 2014; Yang et al., 2020). Eppenberger et al. tested 305 primary breast tumours and found ANG levels to be positively correlated with disease relapse in patients with breast cancer (Eppenberger et al., 1998). Our results, regarding ANG being an independent prognostic factor for OS, PFS, and LRFS are in accordance with the results reported by previous studies. These results collectively recommended ANG as an effective prognostic factor for predicting clinical outcomes in patients with NPC. Since most studies have reported the prognostic significance of ANG on LRFS, PFS, and OS, but not on DMFS, the finding that ANG could not predict DMFS was in accordance with previous studies.

Based on the significant impact of ANG on the survival of patients with NPC and the excellent quantitative ability of the nomogram, a simple and easy-to-use clinical prediction model was established to clinically predict OS in NPC. The nomogram demonstrated favourable discriminative capacity and satisfactory agreement between the predicted and actual results.

There are a few limitations of our study. First, the present study did not explore the exact biological mechanisms underlying ANG-induced radio-resistance. Secondly, the samples of NPC tissues were extracted from a single centre; therefore, further studies incorporating tumour samples from multiple cancer centres should be conducted to validate our results in the future.

In the present study, we found ANG expression to be increased in recurrent NPC tissues. Elevated expression of ANG induced radio-resistance in NPC cells, in addition to being significantly associated with shorter PFS, OS, and LRFS in patients with NPC. Multivariate analysis results revealed that ANG was an independent prognostic factor that predicted PFS, OS, and LRFS. Furthermore, we designed a nomogram model to predict OS in terms of ANG expression. Our results provided a new perspective on the therapeutic strategies of NPC; it could help predict radio-sensitivity and stratify high-risk patients or tumour recurrence.

## Data Availability

The raw data supporting the conclusion of this article will be made available by the authors, without undue reservation. The authenticity of this article has been validated by uploading the key raw data onto the Research Data Deposit public platform (www.researchdata.org.cn), with the approval RDD number as RDDB2020000965.

## Supplementary Material

Supplementary Figure S1 **(A)** Typical flow cytometry of HONE1 cells cycle arrest, **(B)** apoptosis analysis by flow cytometry of HONE1 cells. **(C)** Transwell assay showed the cell migration of HONE1 cells. **(D)** The relative mRNA expression of HONE1 cells.

## Abbreviations

ANG, angiogenin; CCRT, concurrent chemoradiotherapy; PFS, progression-free survival; DMFS, distant metastasis-free survival; FBS, foetal bovine serum; IC, induction chemoradiotherapy; IHC, immunohistochemistry; IMRT, intensity-modulated radiotherapy; IV, intravenous; LRFS, locoregional recurrence-free survival; NPC, nasopharyngeal carcinoma; OS, overall survival; PFS, progression-free survival; RT, radiotherapy; SDS, sodium dodecyl sulphate; siRNA, small interfering RNA.
