# Supplementary figures and images for: Increased Angiogenin Expression Correlates With Radiation Resistance and Predicts Poor Survival for Patients With Nasopharyngeal Carcinoma
Source: Front Pharmacol. 2021 Aug 26;12:627935. doi: 10.3389/fphar.2021.627935 (PMC8427601; doi:10.3389/fphar.2021.627935)

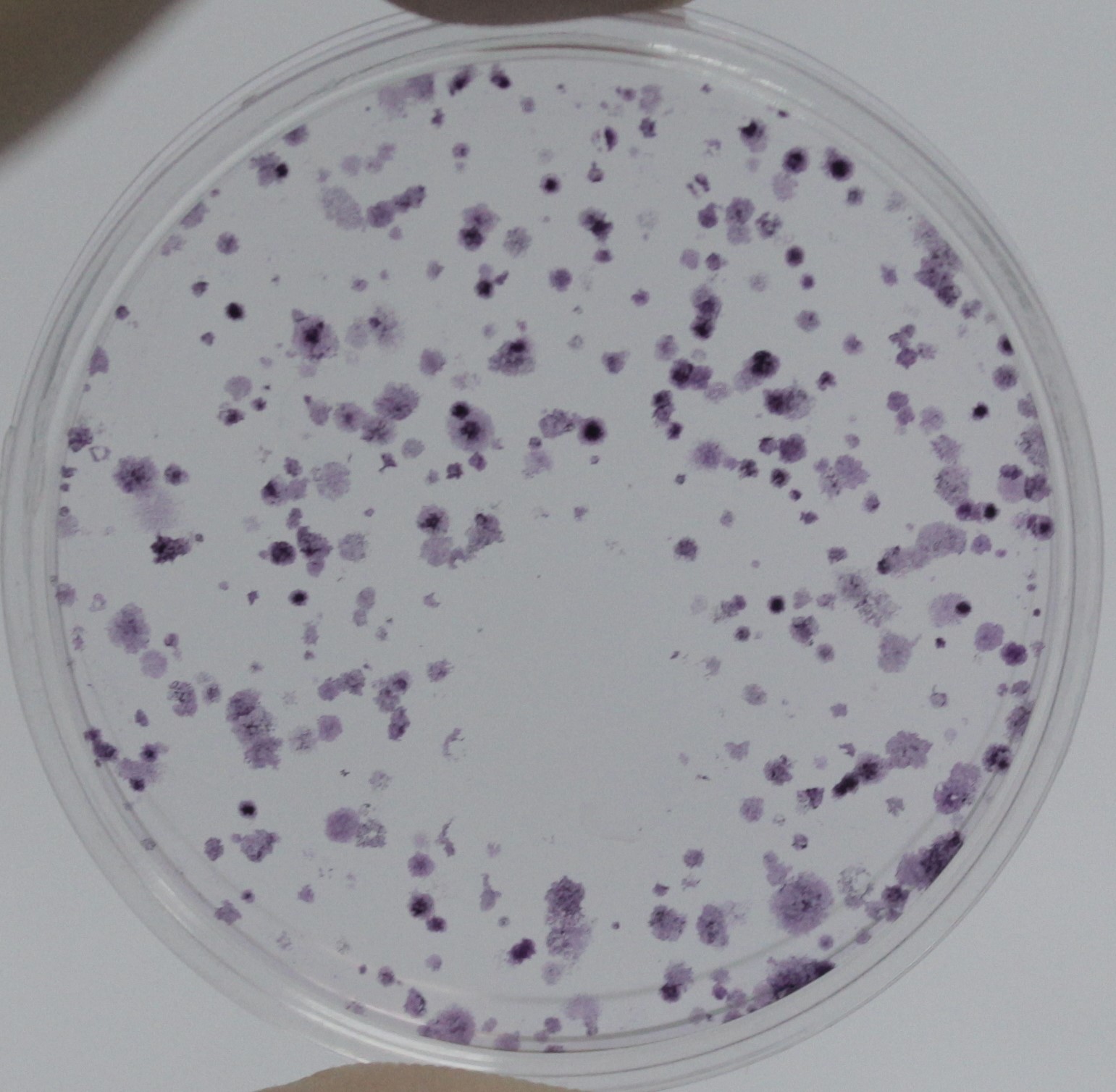

Supplement: Supplementary file 1 [file DataSheet1.zip › figure1data/figure1C-radioresitant function of siANG and HONE-IR/HONE1IR-0Gy.JPG]

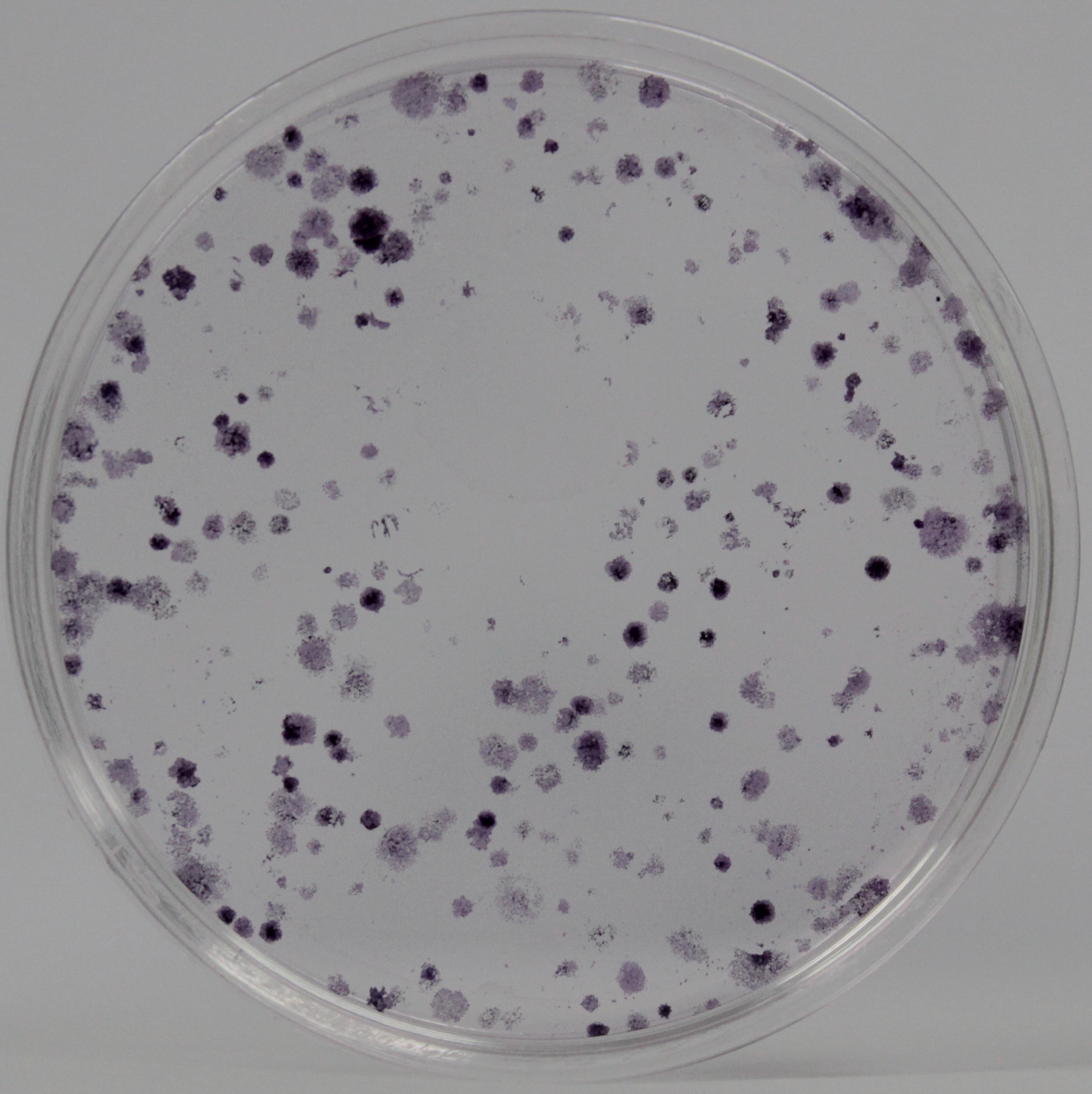

Supplement: Supplementary file 1 [file DataSheet1.zip › figure1data/figure1C-radioresitant function of siANG and HONE-IR/HONE1IR-1Gy.JPG]

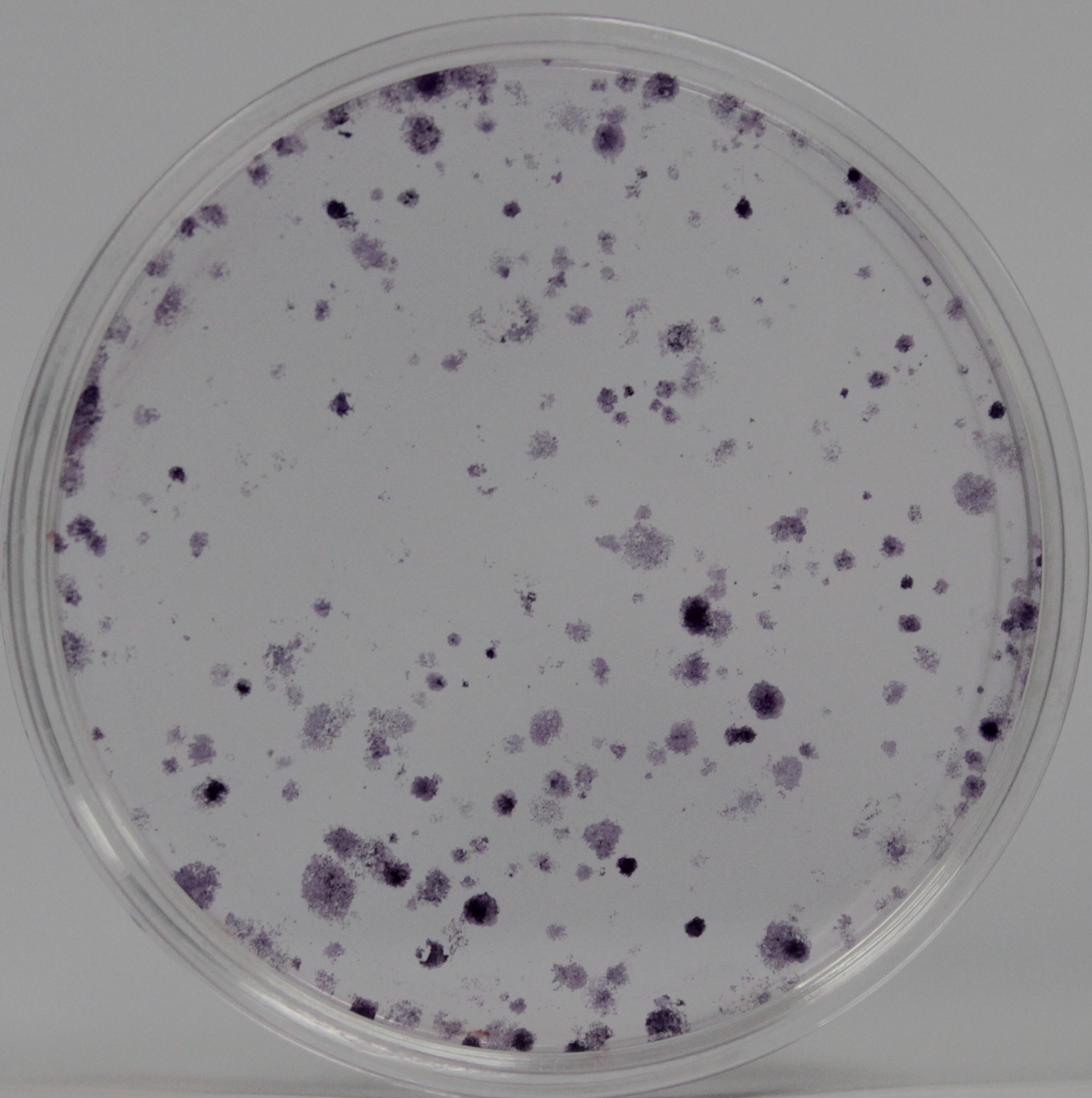

Supplement: Supplementary file 1 [file DataSheet1.zip › figure1data/figure1C-radioresitant function of siANG and HONE-IR/HONE1IR-2Gy.JPG]

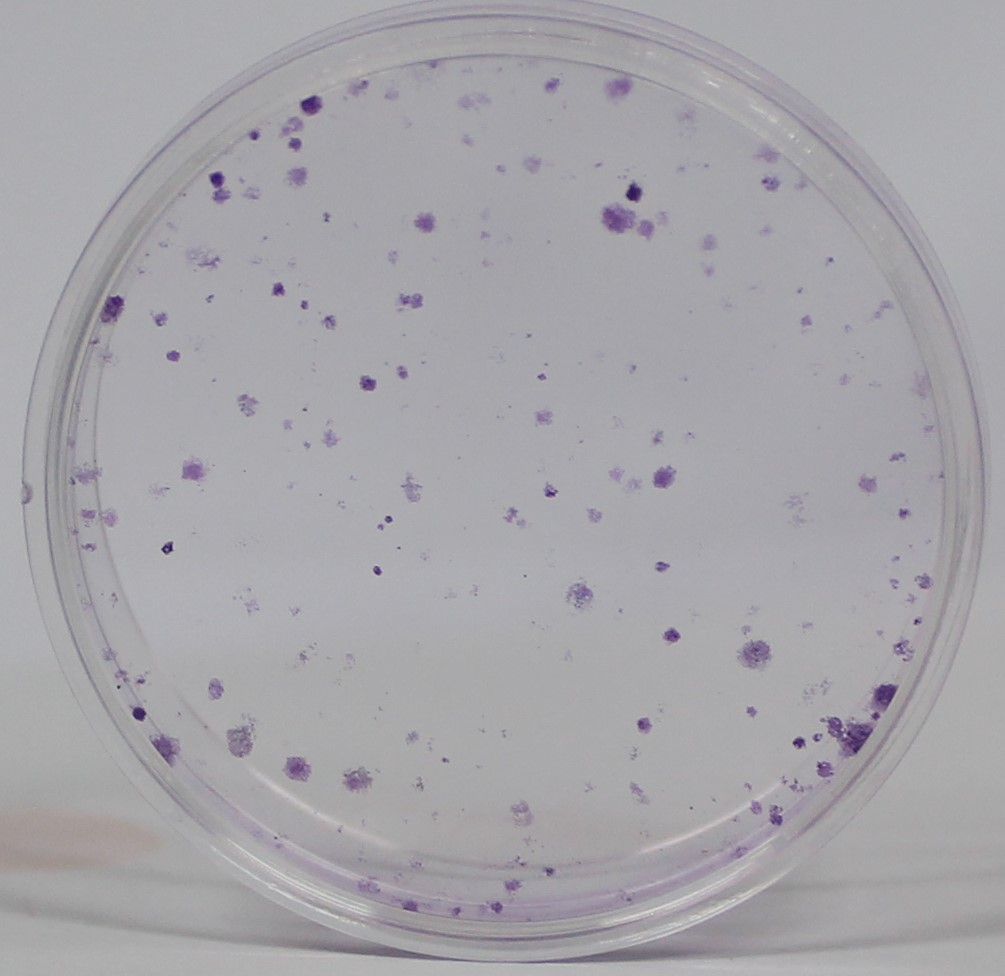

Supplement: Supplementary file 1 [file DataSheet1.zip › figure1data/figure1C-radioresitant function of siANG and HONE-IR/HONE1IR-3Gy.JPG]

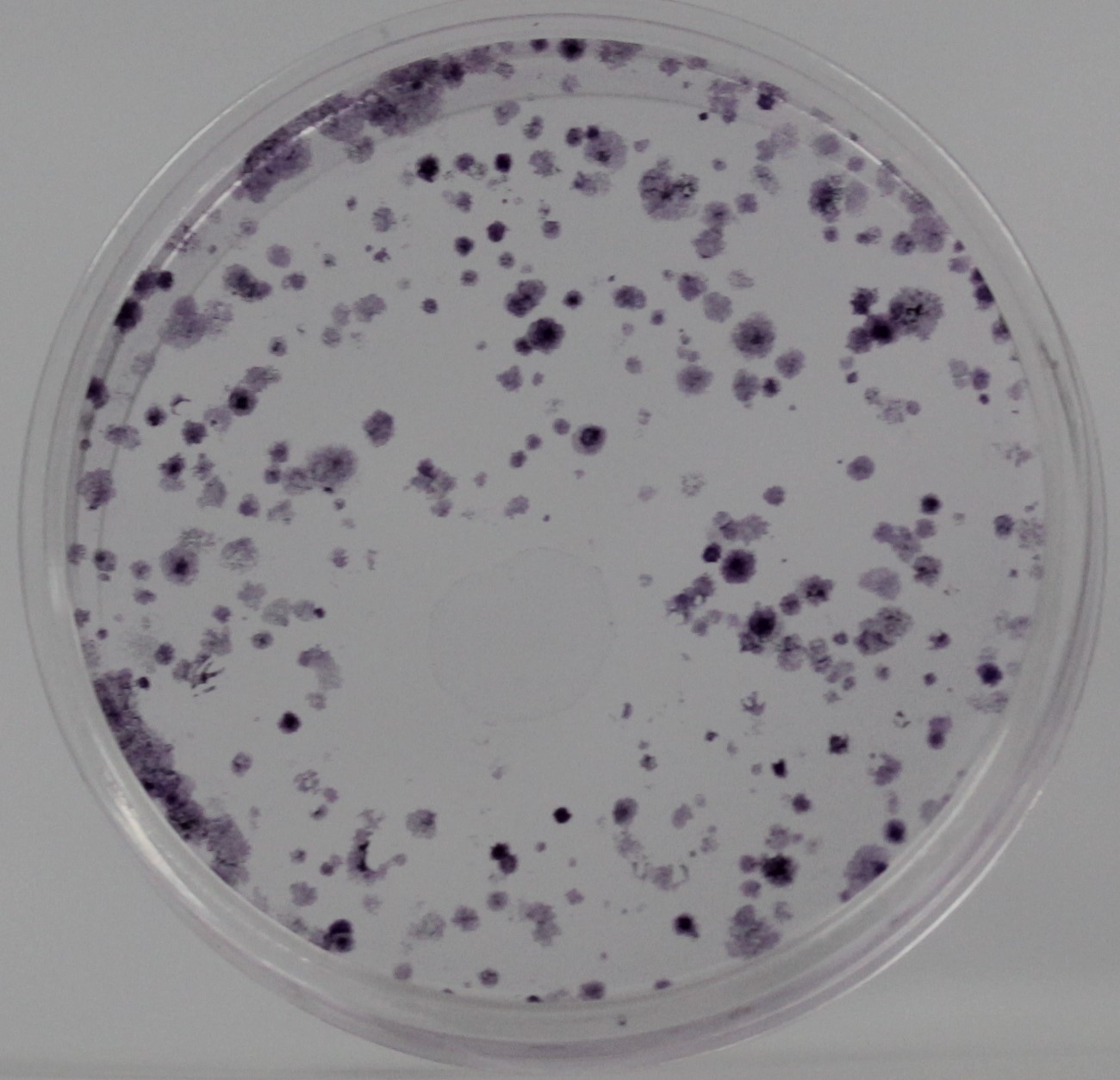

Supplement: Supplementary file 1 [file DataSheet1.zip › figure1data/figure1C-radioresitant function of siANG and HONE-IR/siANG-0Gy.JPG]

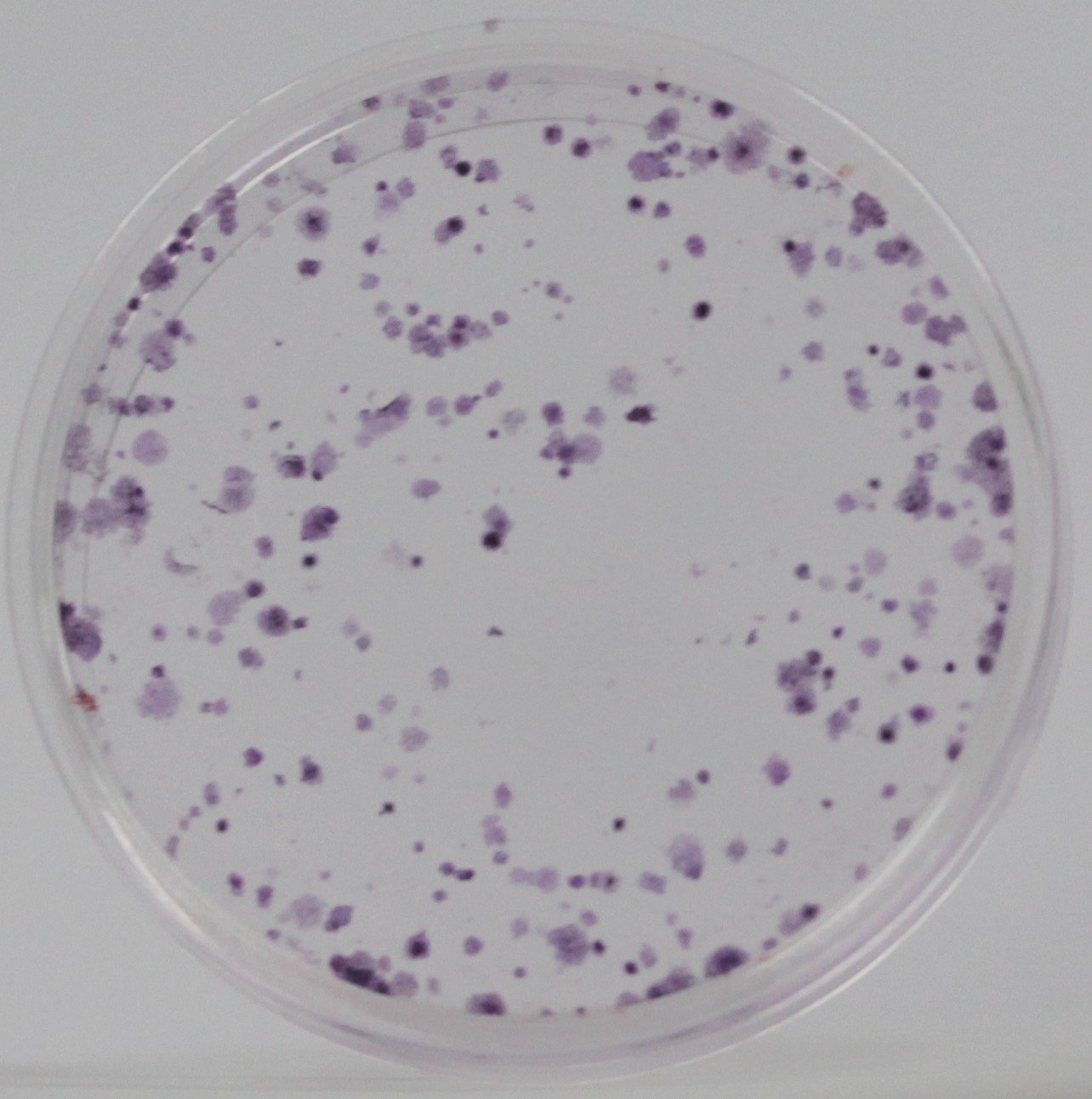

Supplement: Supplementary file 1 [file DataSheet1.zip › figure1data/figure1C-radioresitant function of siANG and HONE-IR/siANG-1Gy.JPG]

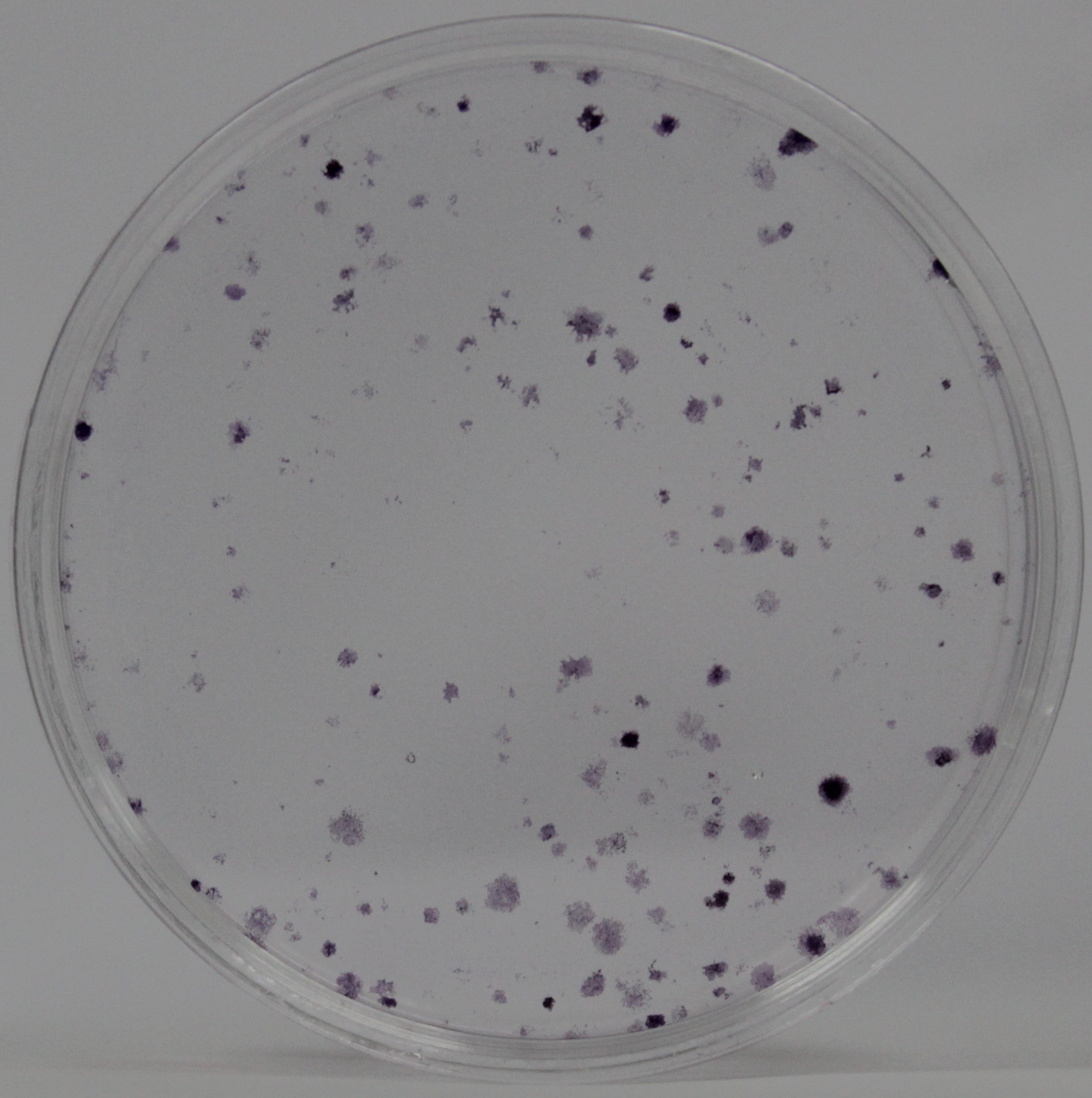

Supplement: Supplementary file 1 [file DataSheet1.zip › figure1data/figure1C-radioresitant function of siANG and HONE-IR/siANG-2Gy.JPG]

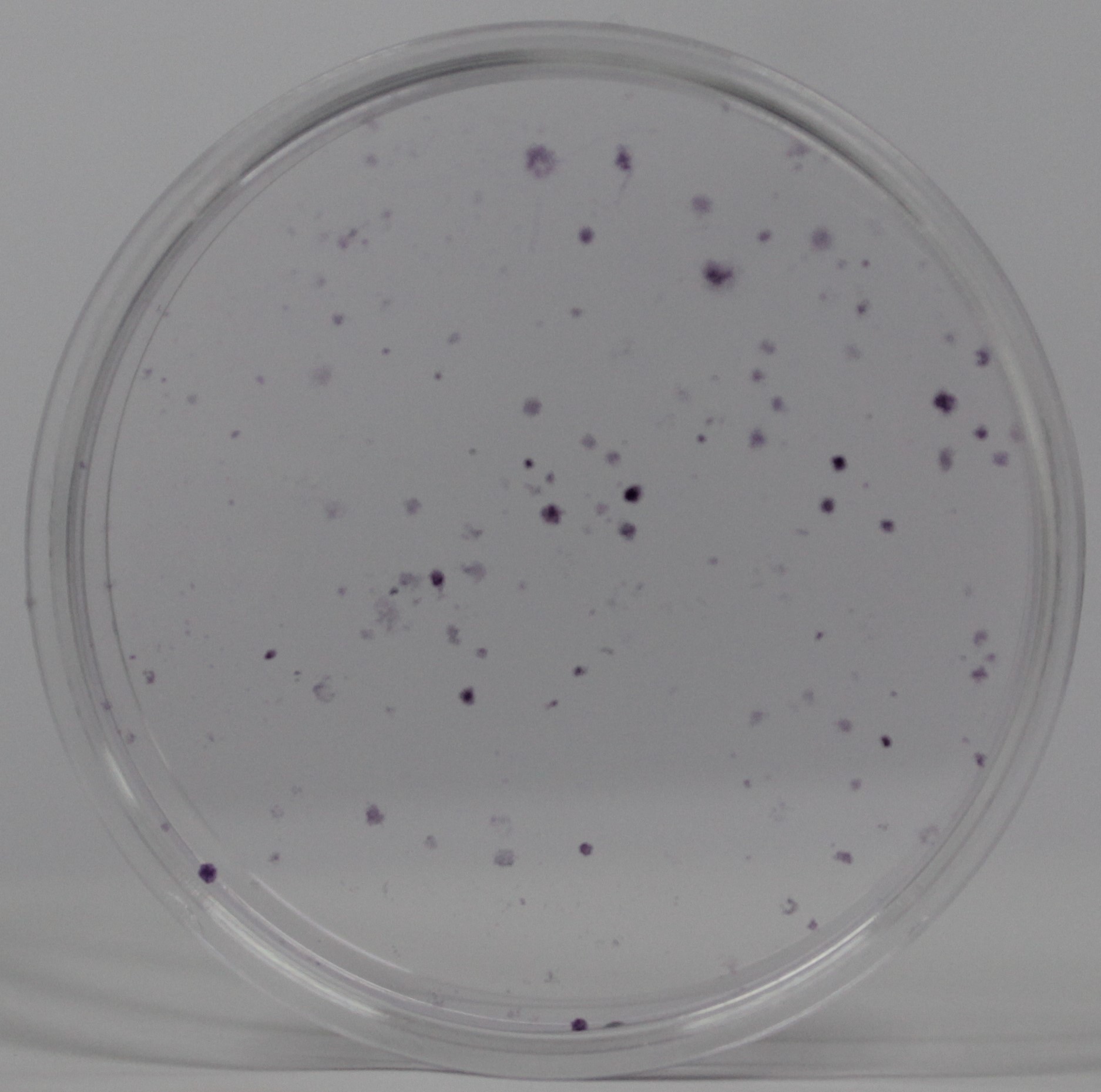

Supplement: Supplementary file 1 [file DataSheet1.zip › figure1data/figure1C-radioresitant function of siANG and HONE-IR/siANG-3Gy.JPG]

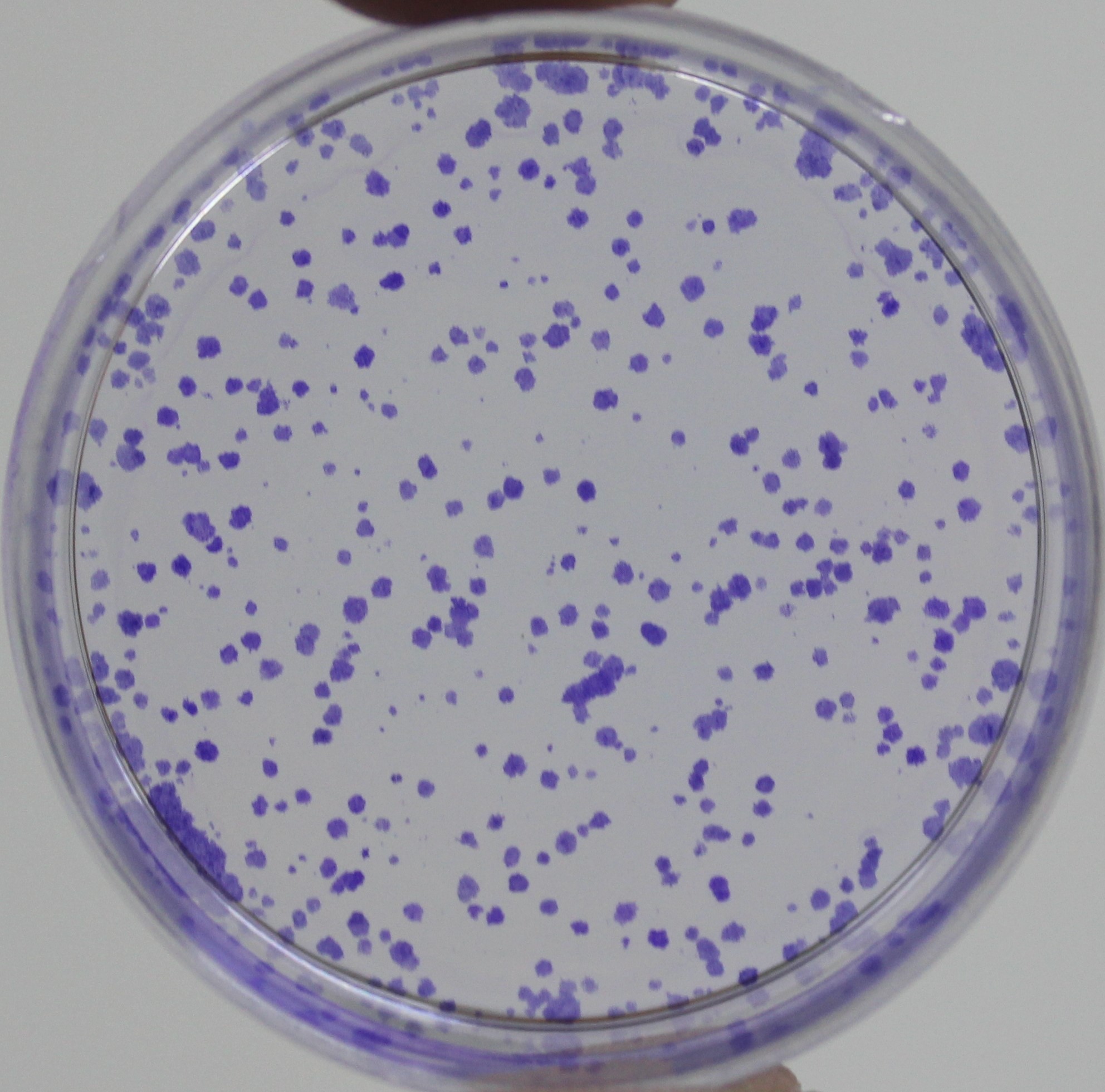

Supplement: Supplementary file 1 [file DataSheet1.zip › figure1data/figure1C-raioresistant function of rhpANG and control/Control-0Gy.JPG]

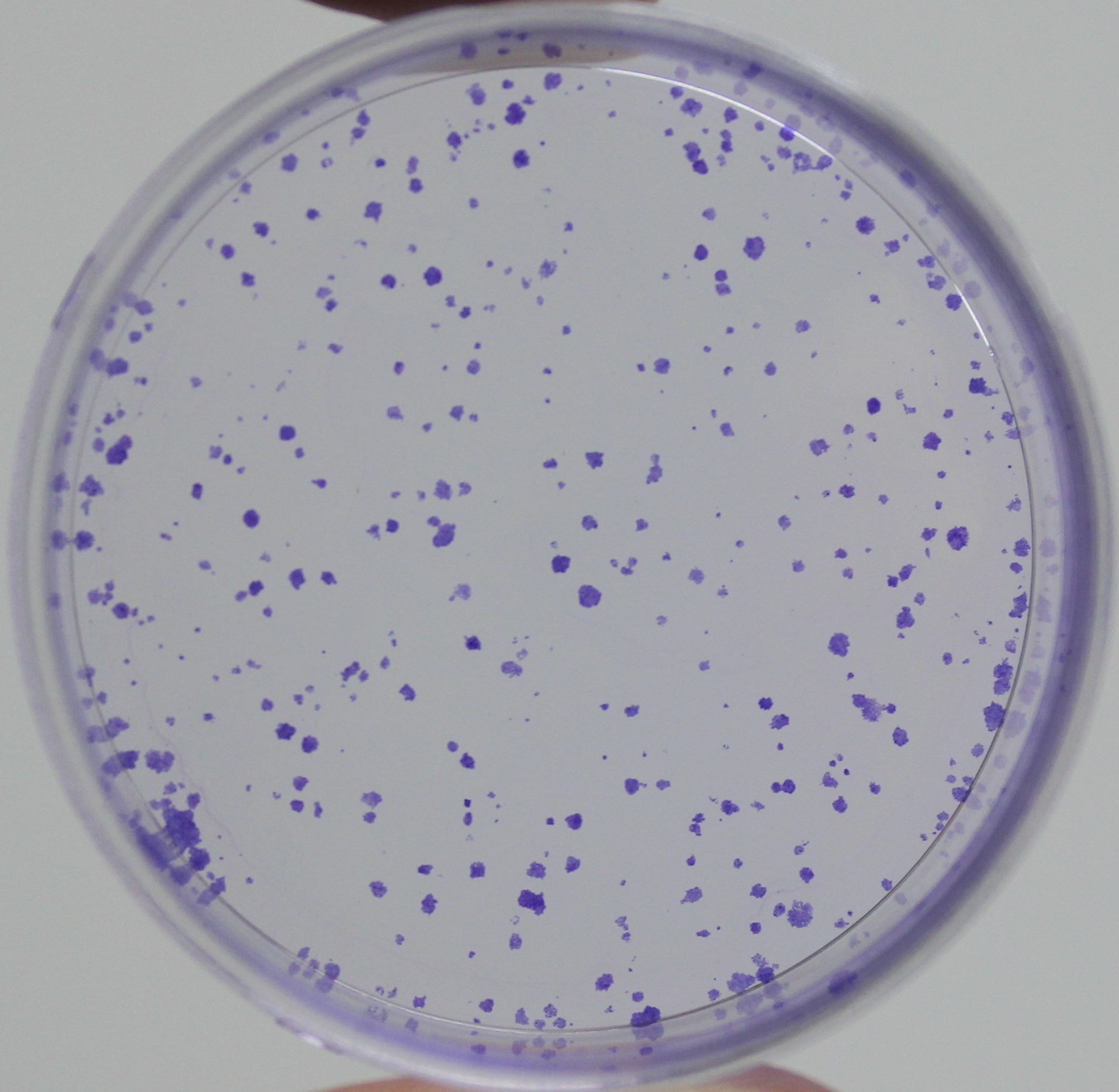

Supplement: Supplementary file 1 [file DataSheet1.zip › figure1data/figure1C-raioresistant function of rhpANG and control/Control-1Gy.JPG]

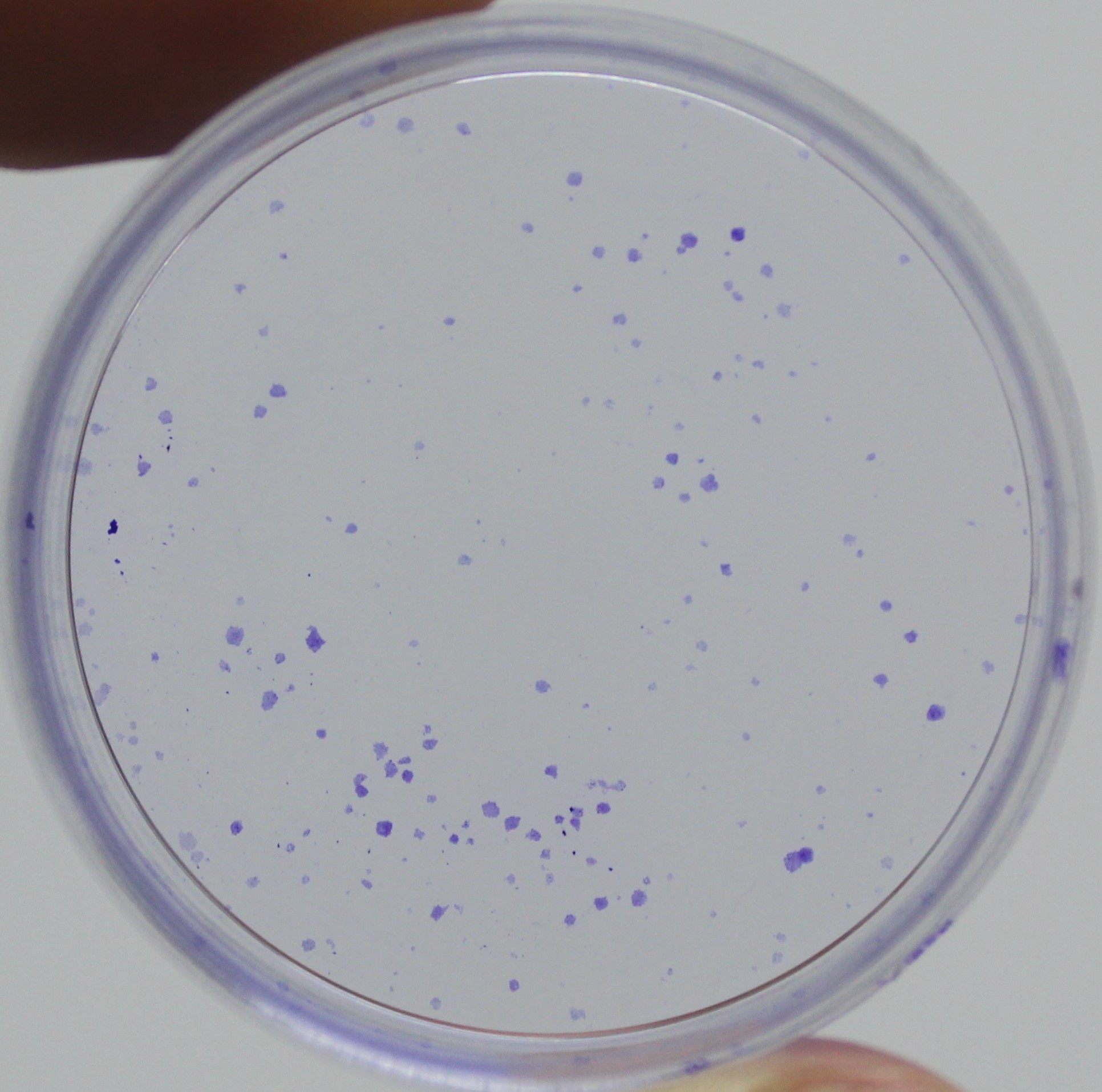

Supplement: Supplementary file 1 [file DataSheet1.zip › figure1data/figure1C-raioresistant function of rhpANG and control/Control-2Gy.JPG]

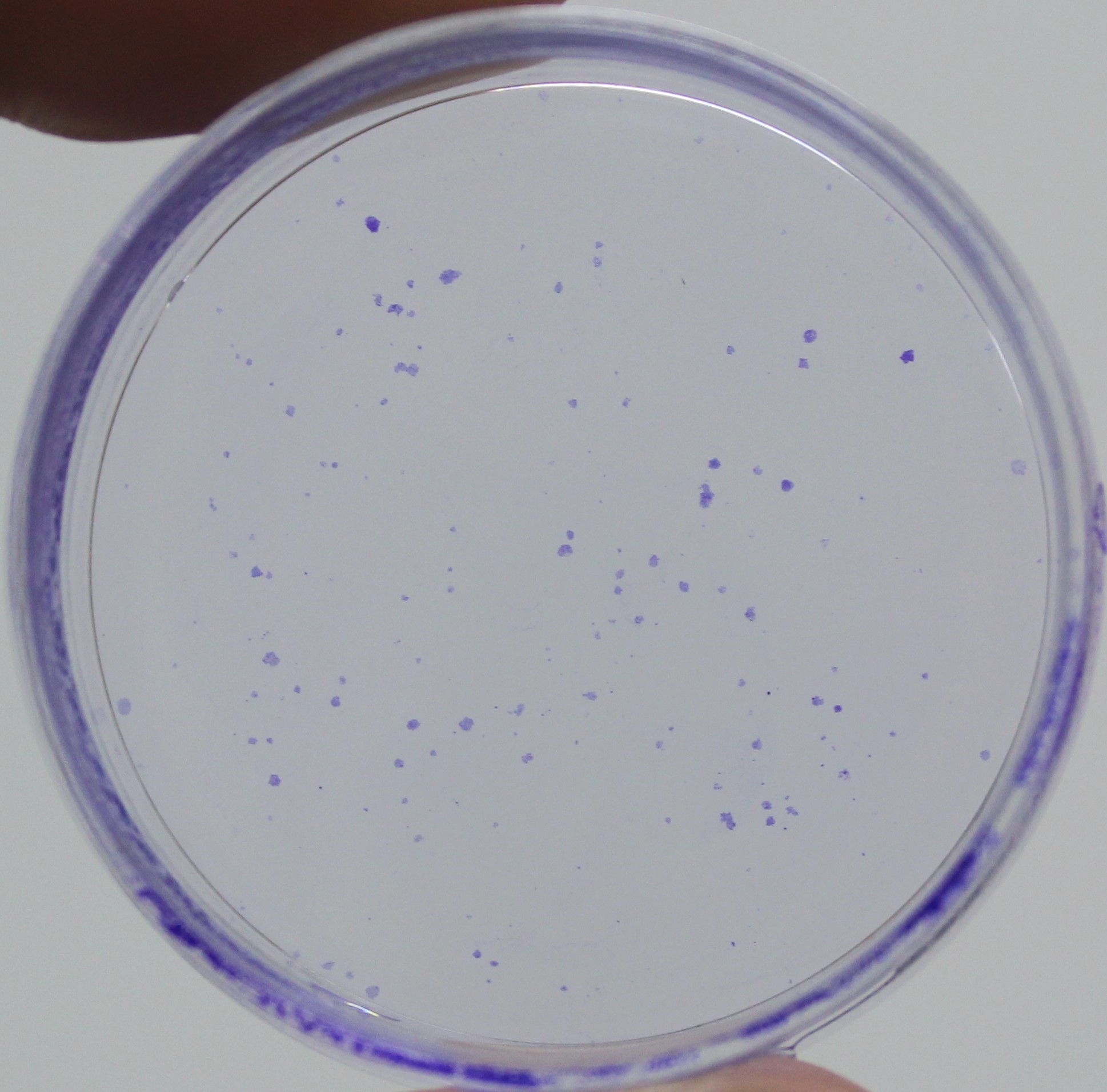

Supplement: Supplementary file 1 [file DataSheet1.zip › figure1data/figure1C-raioresistant function of rhpANG and control/Control-3Gy.JPG]

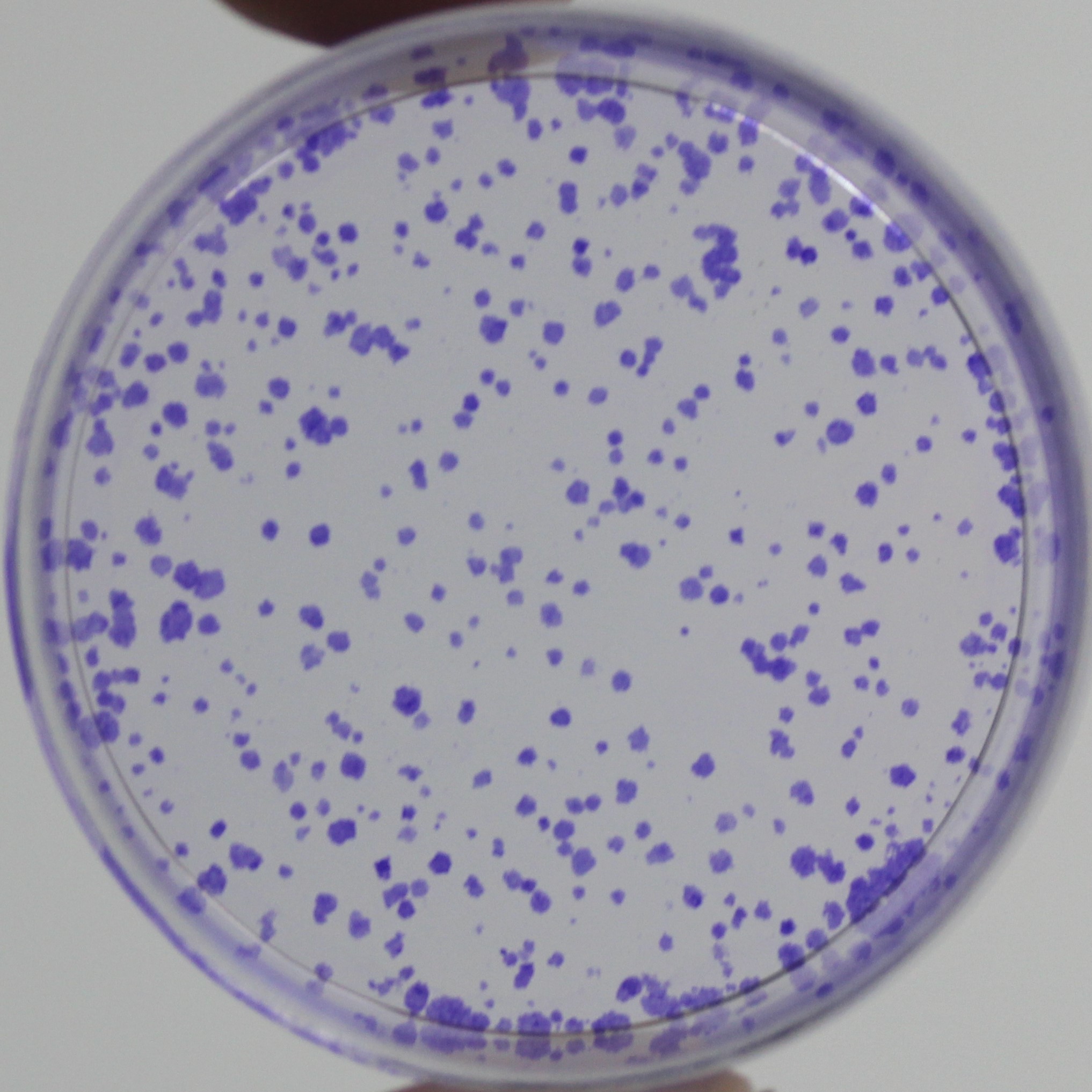

Supplement: Supplementary file 1 [file DataSheet1.zip › figure1data/figure1C-raioresistant function of rhpANG and control/hrpANG-0Gy.JPG]

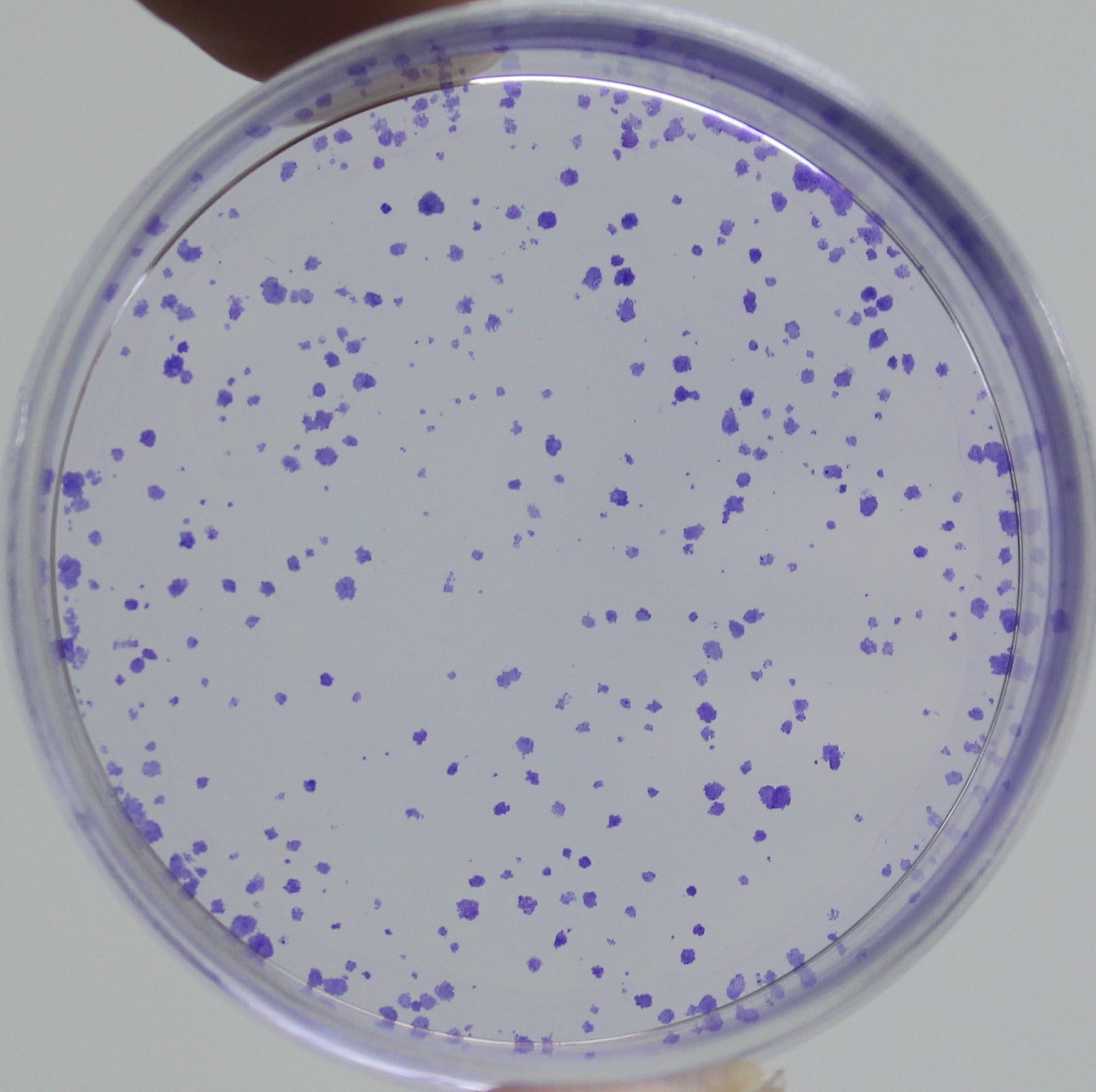

Supplement: Supplementary file 1 [file DataSheet1.zip › figure1data/figure1C-raioresistant function of rhpANG and control/hrpANG-1Gy.JPG]

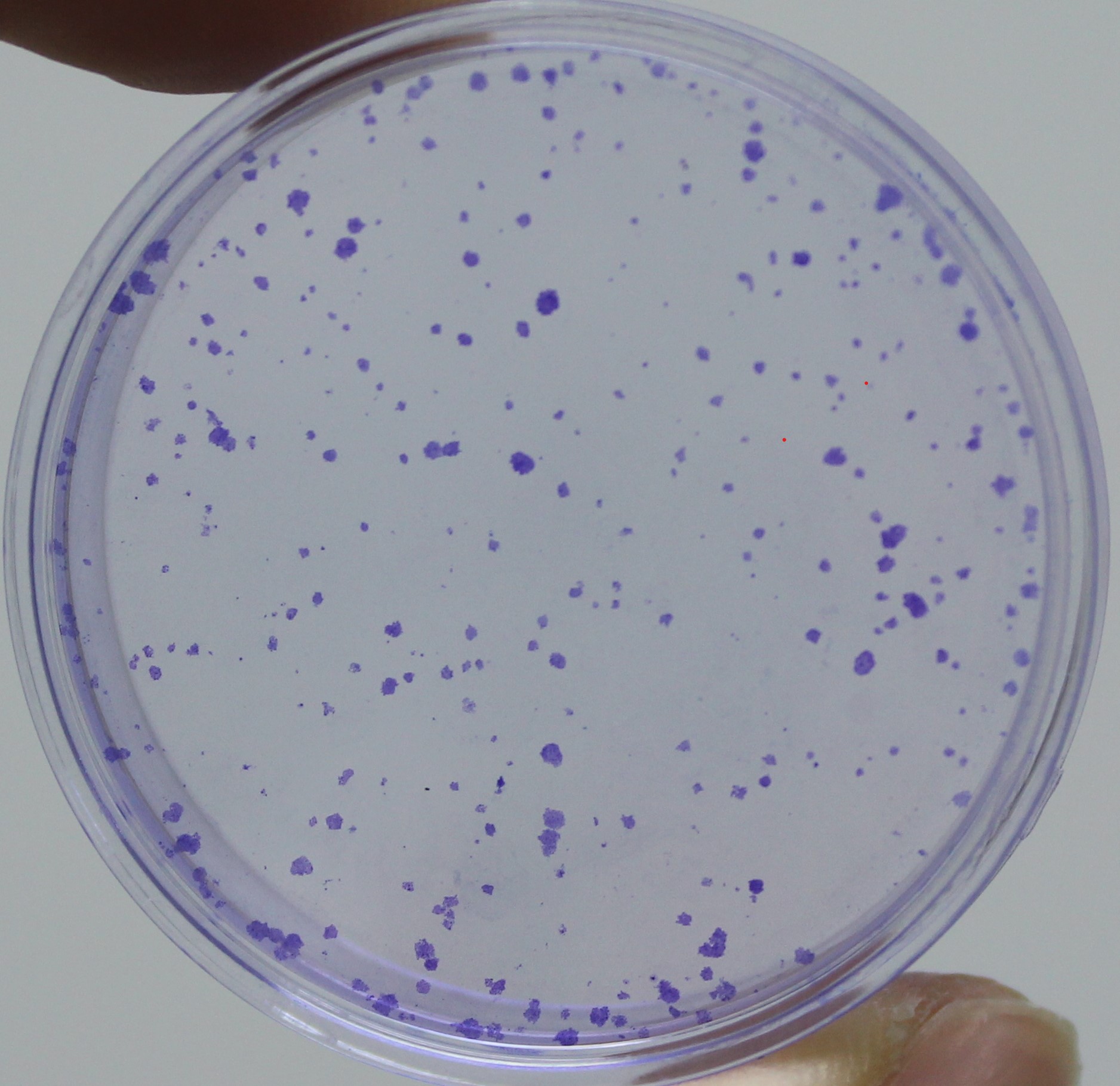

Supplement: Supplementary file 1 [file DataSheet1.zip › figure1data/figure1C-raioresistant function of rhpANG and control/hrpANG-2Gy.JPG]

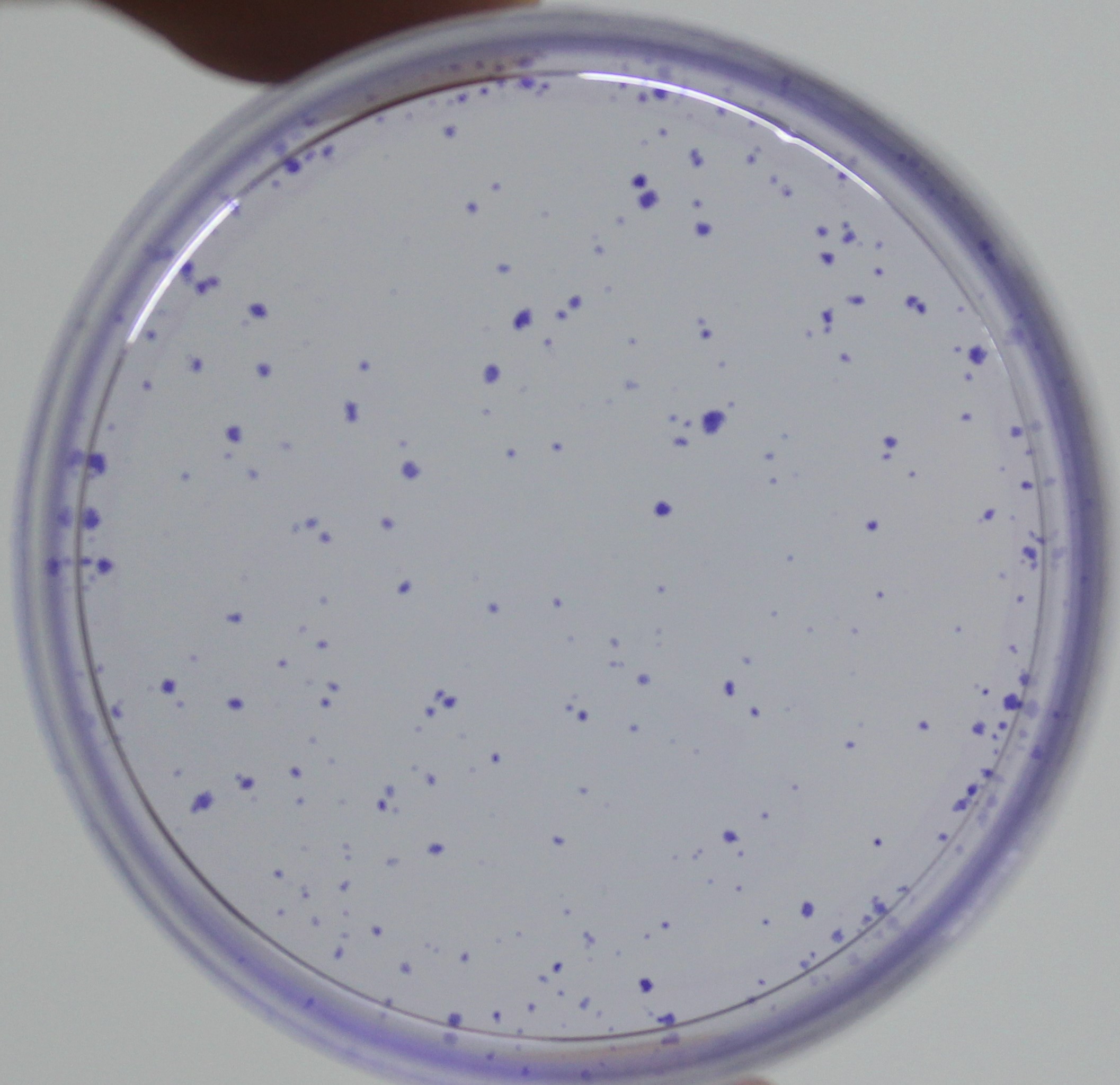

Supplement: Supplementary file 1 [file DataSheet1.zip › figure1data/figure1C-raioresistant function of rhpANG and control/hrpANG-3Gy.JPG]

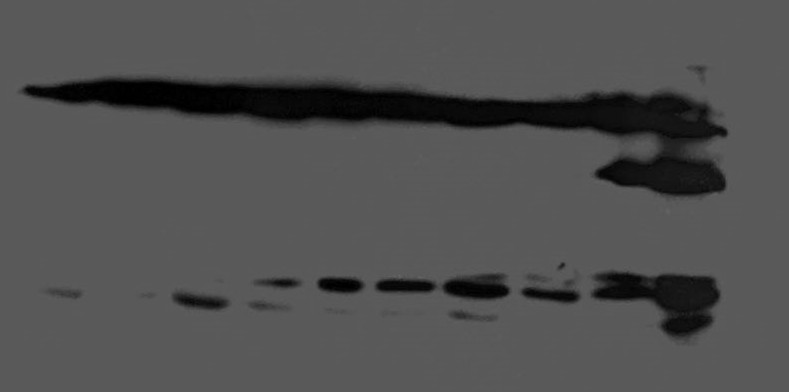

Supplement: Supplementary file 1 [file DataSheet1.zip › figure1data/figure1D-western blot (1).jpg]

Actual 3-year OS (proportion)

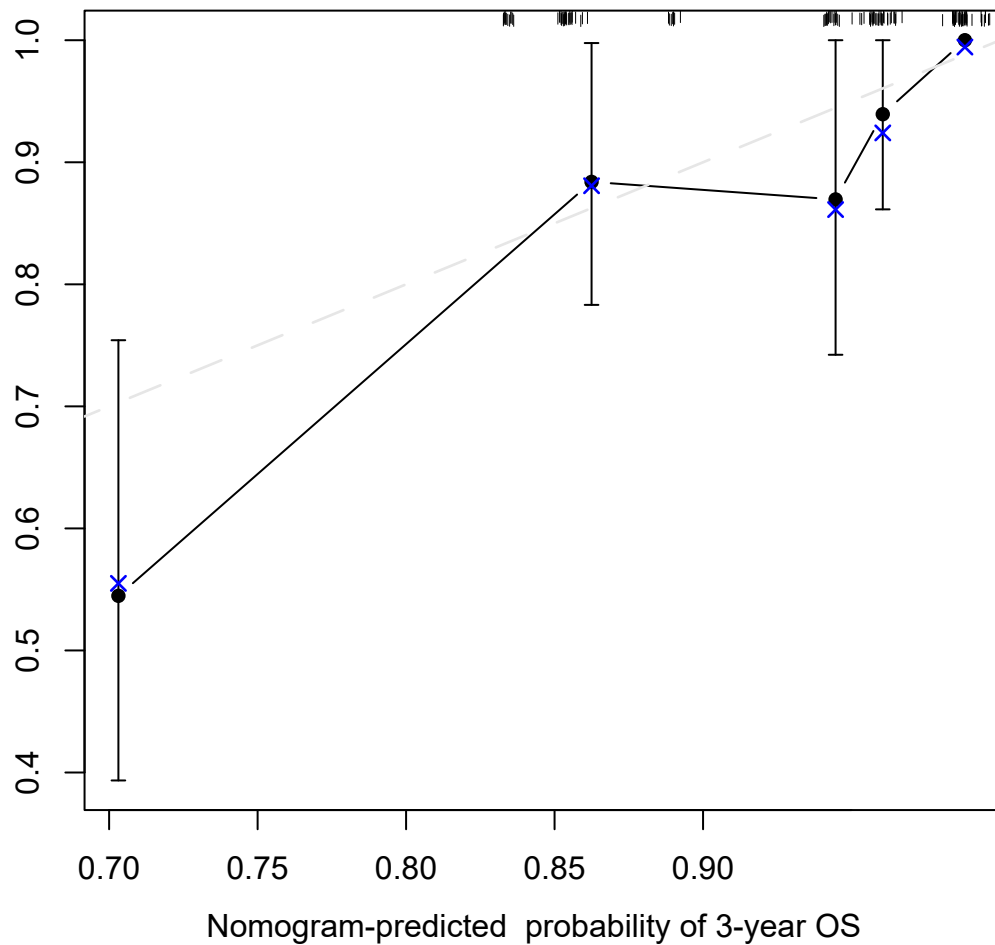

Actual 5-year OS (proportion)

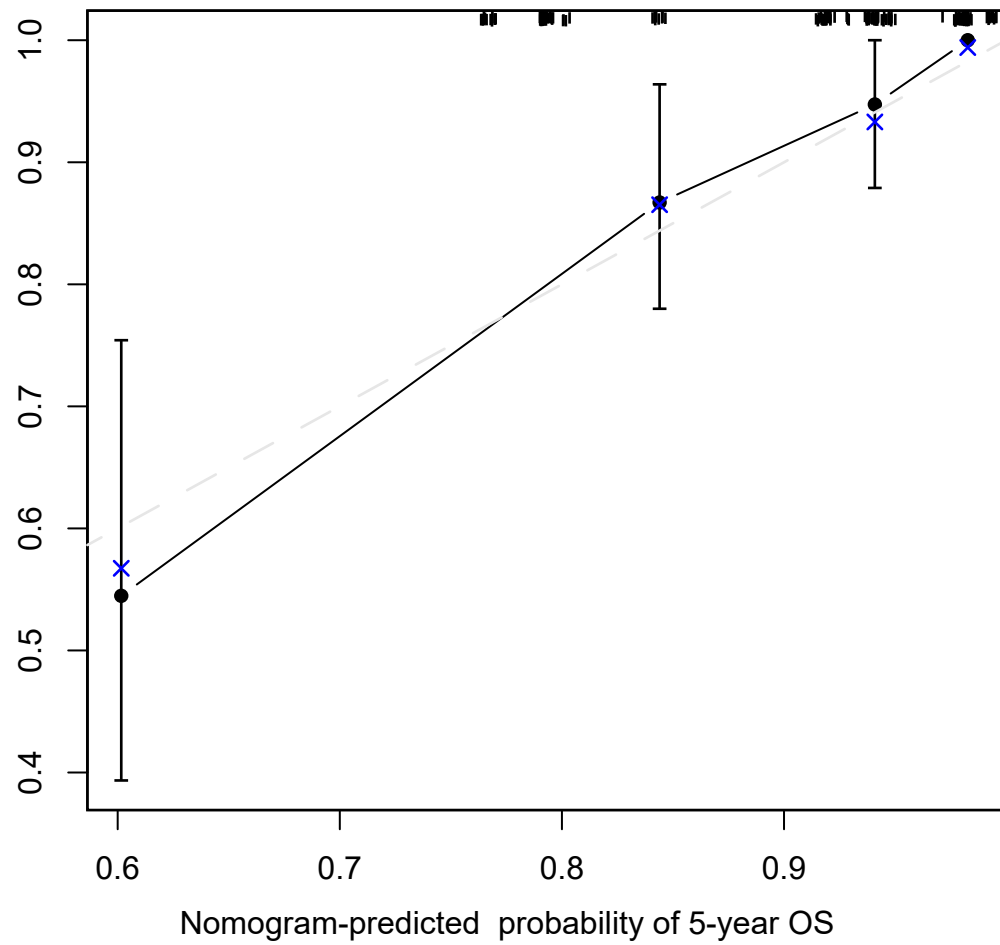

Supplement: Supplementary file 1 [file DataSheet1.zip › figure3 table1 table2-source data/figure3-calibration_curves of nomogram model.pdf]

Points

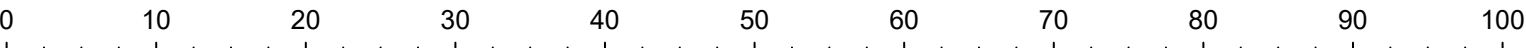

T

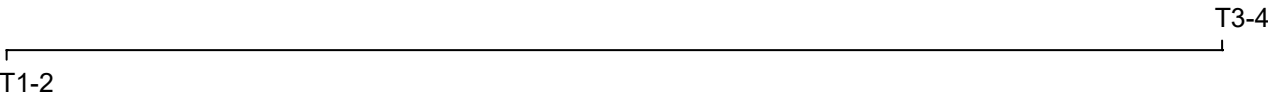

N

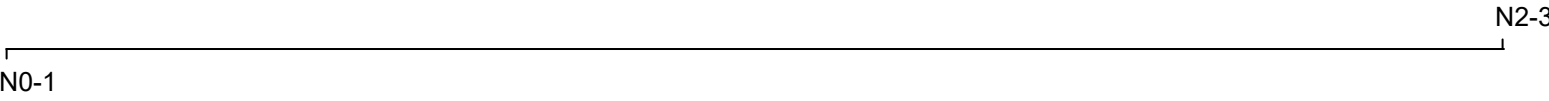

EBVDNA

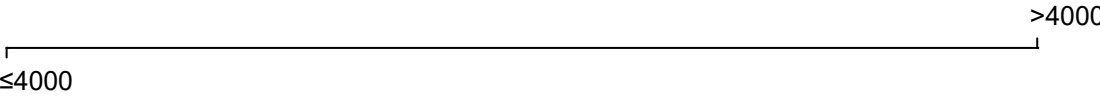

ANG

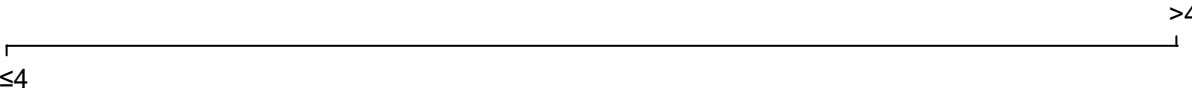

Total Points

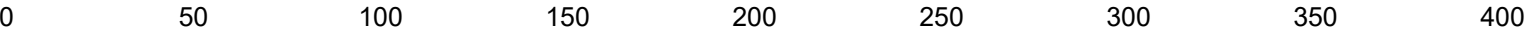

Linear Predictor

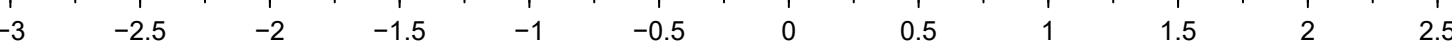

3 year survival

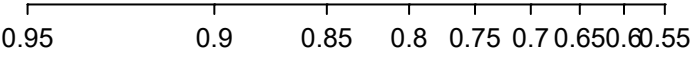

5 year survival

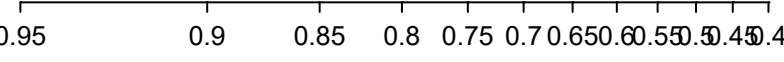

Supplement: Supplementary file 1 [file DataSheet1.zip › figure3 table1 table2-source data/figure3-Nomogram.pdf]

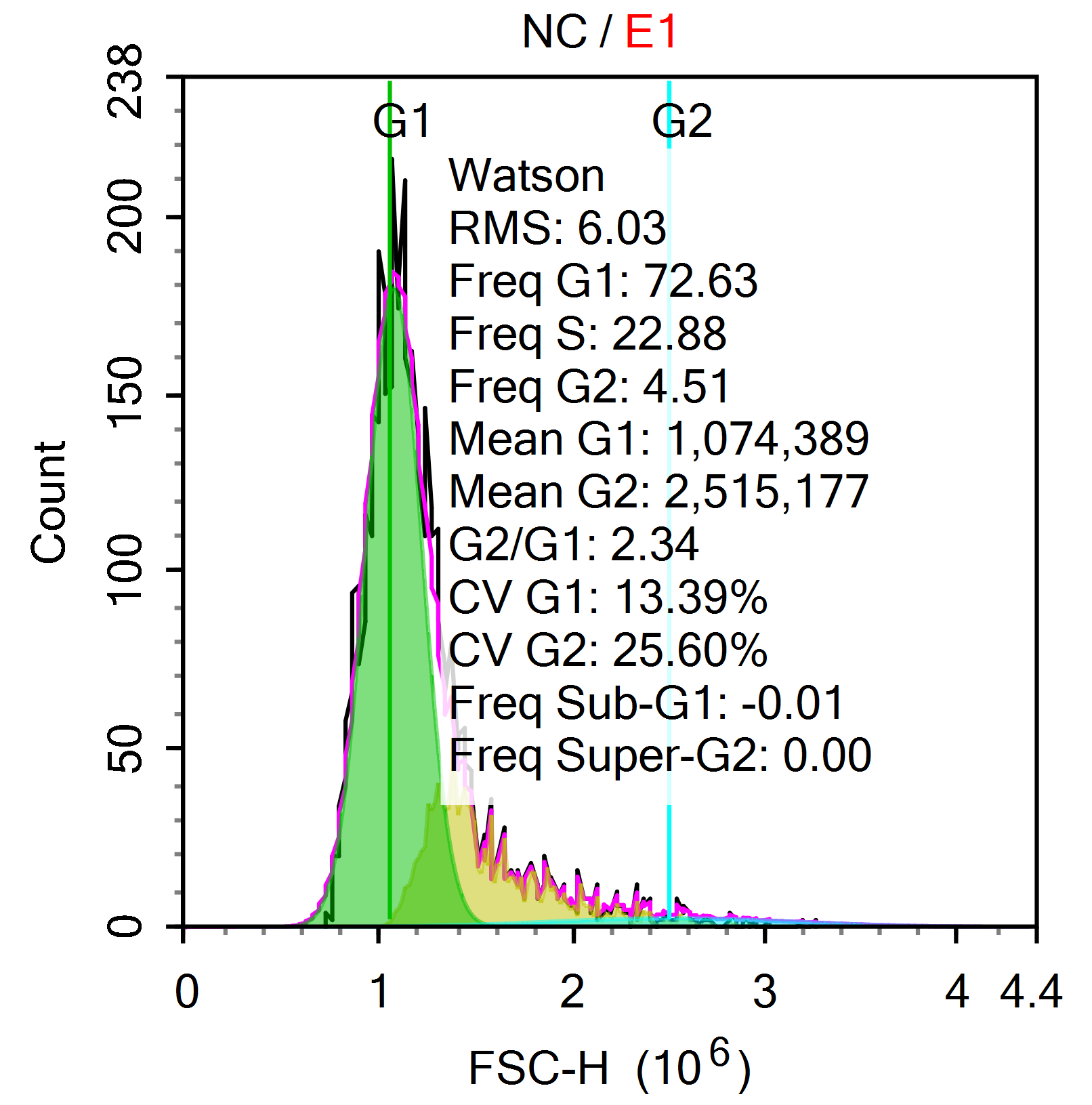

Supplement: Supplementary file 1 [file DataSheet1.zip › supplementary figure1 data/cell cycle/nc/base on fitc cell cycle analysis.tiff]

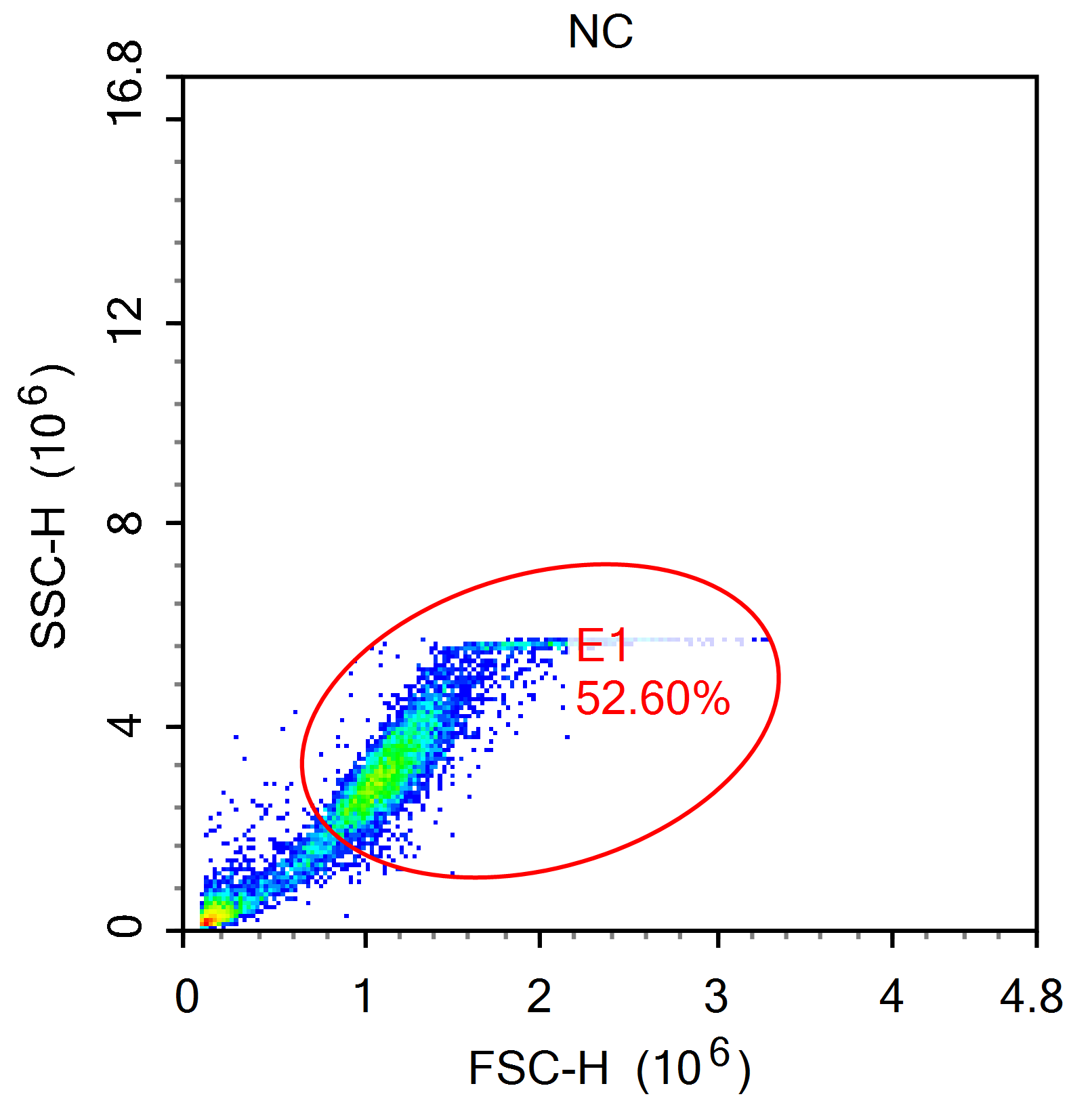

Supplement: Supplementary file 1 [file DataSheet1.zip › supplementary figure1 data/cell cycle/nc/FCS and ssc analysis.tiff]

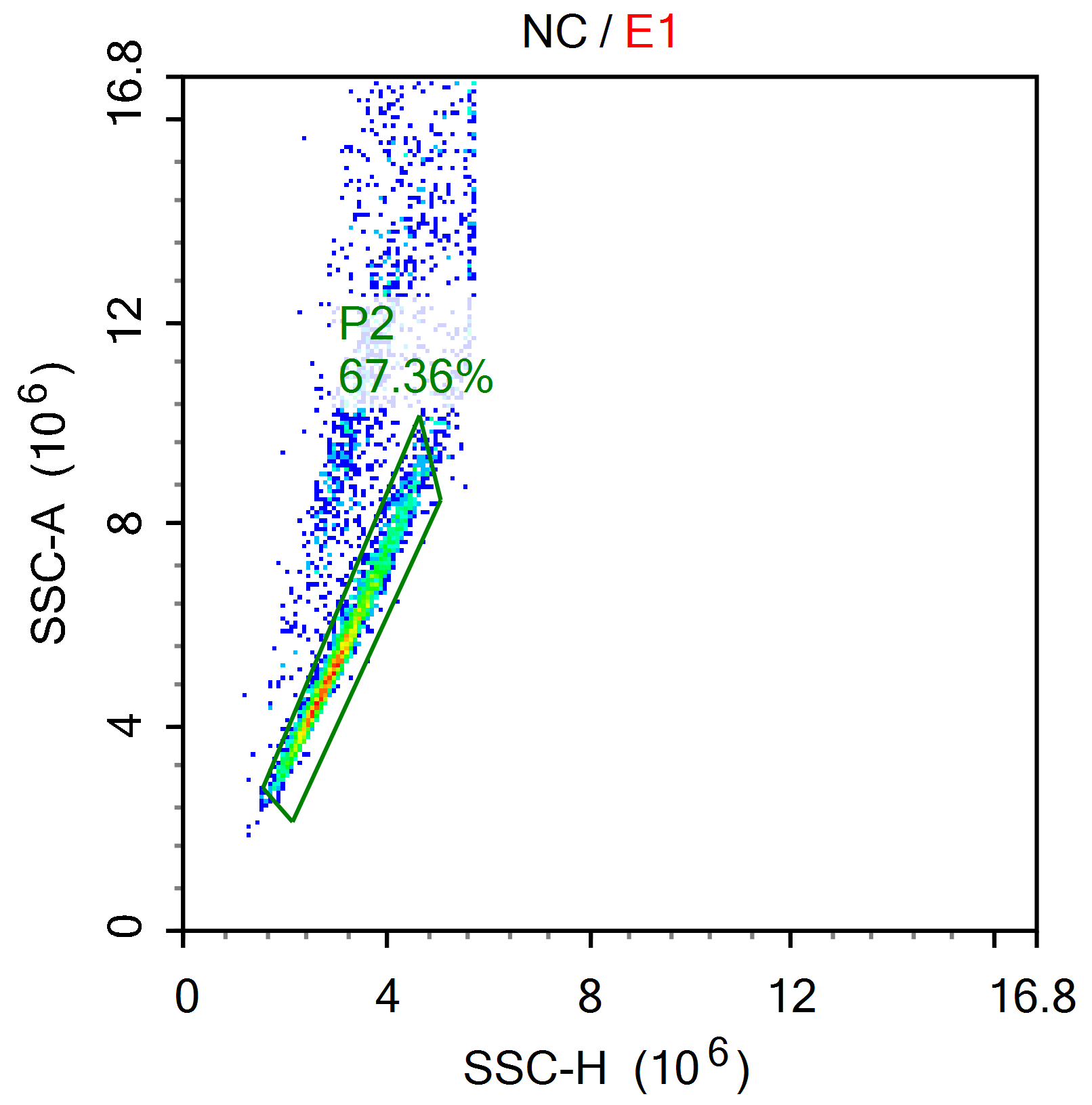

Supplement: Supplementary file 1 [file DataSheet1.zip › supplementary figure1 data/cell cycle/nc/Remove diploid analysis.tiff]

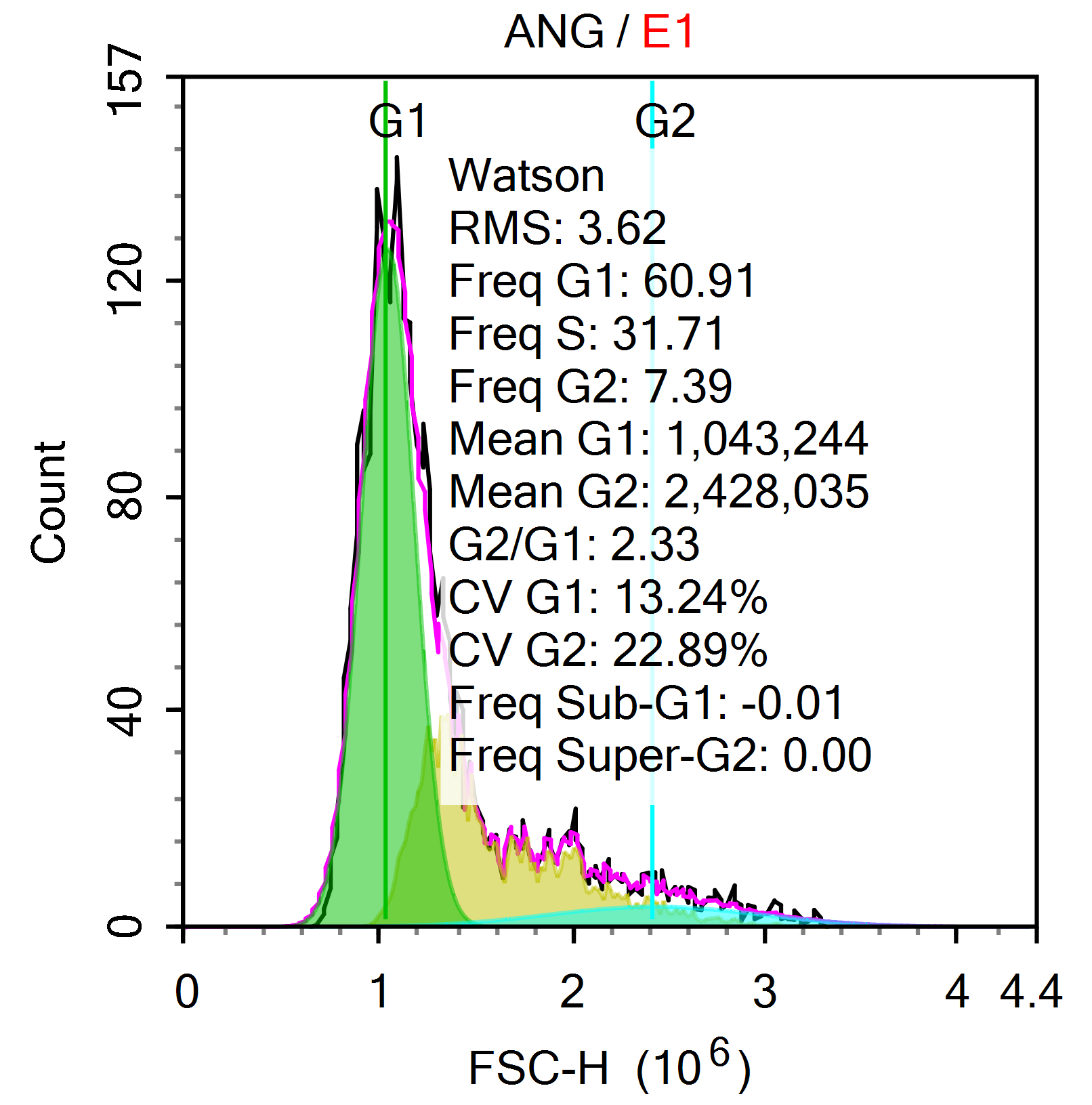

Supplement: Supplementary file 1 [file DataSheet1.zip › supplementary figure1 data/cell cycle/siANG/base on fitc cell cycle analysis.tiff]

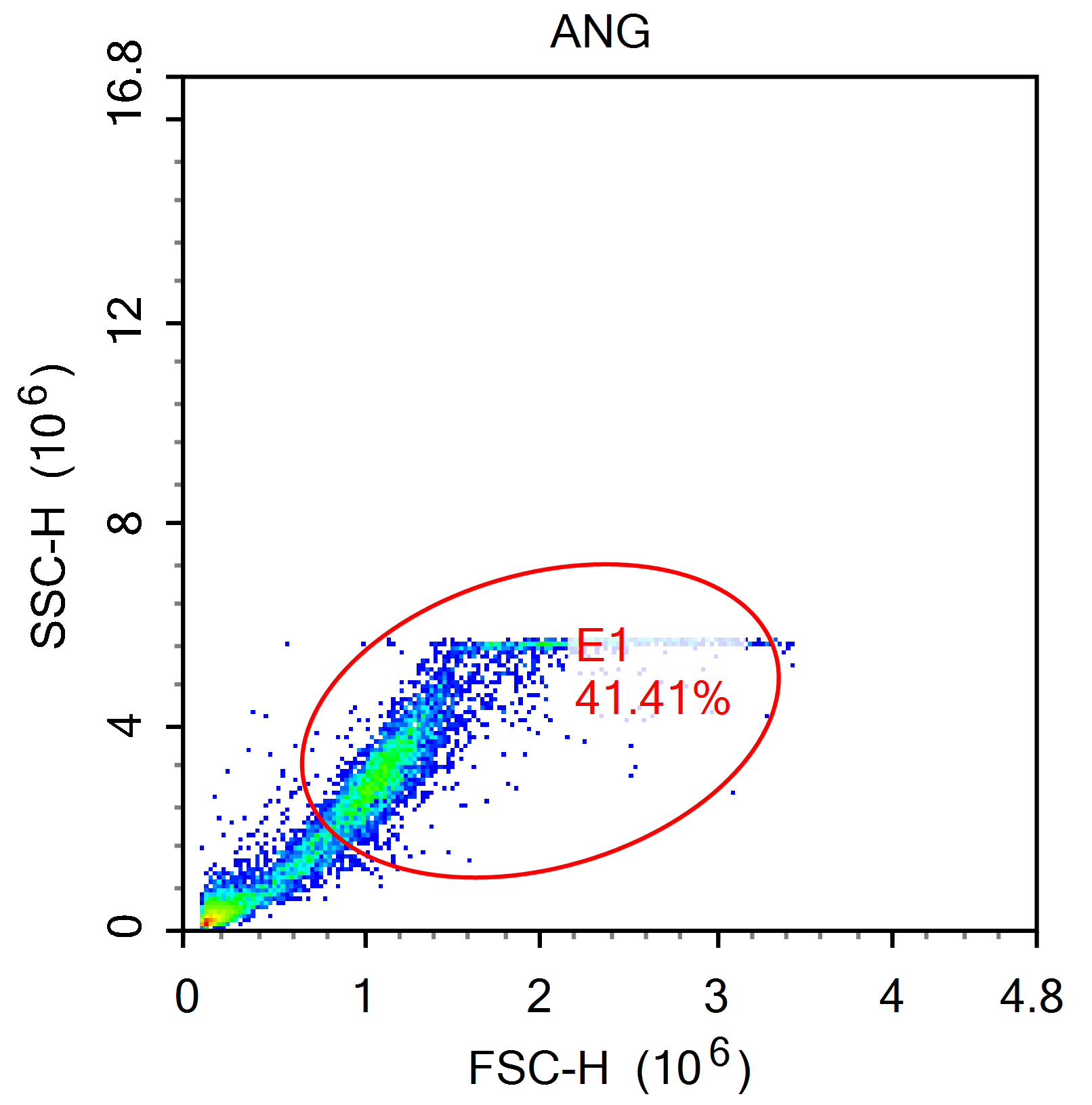

Supplement: Supplementary file 1 [file DataSheet1.zip › supplementary figure1 data/cell cycle/siANG/FCS and ssc analysis.tiff]

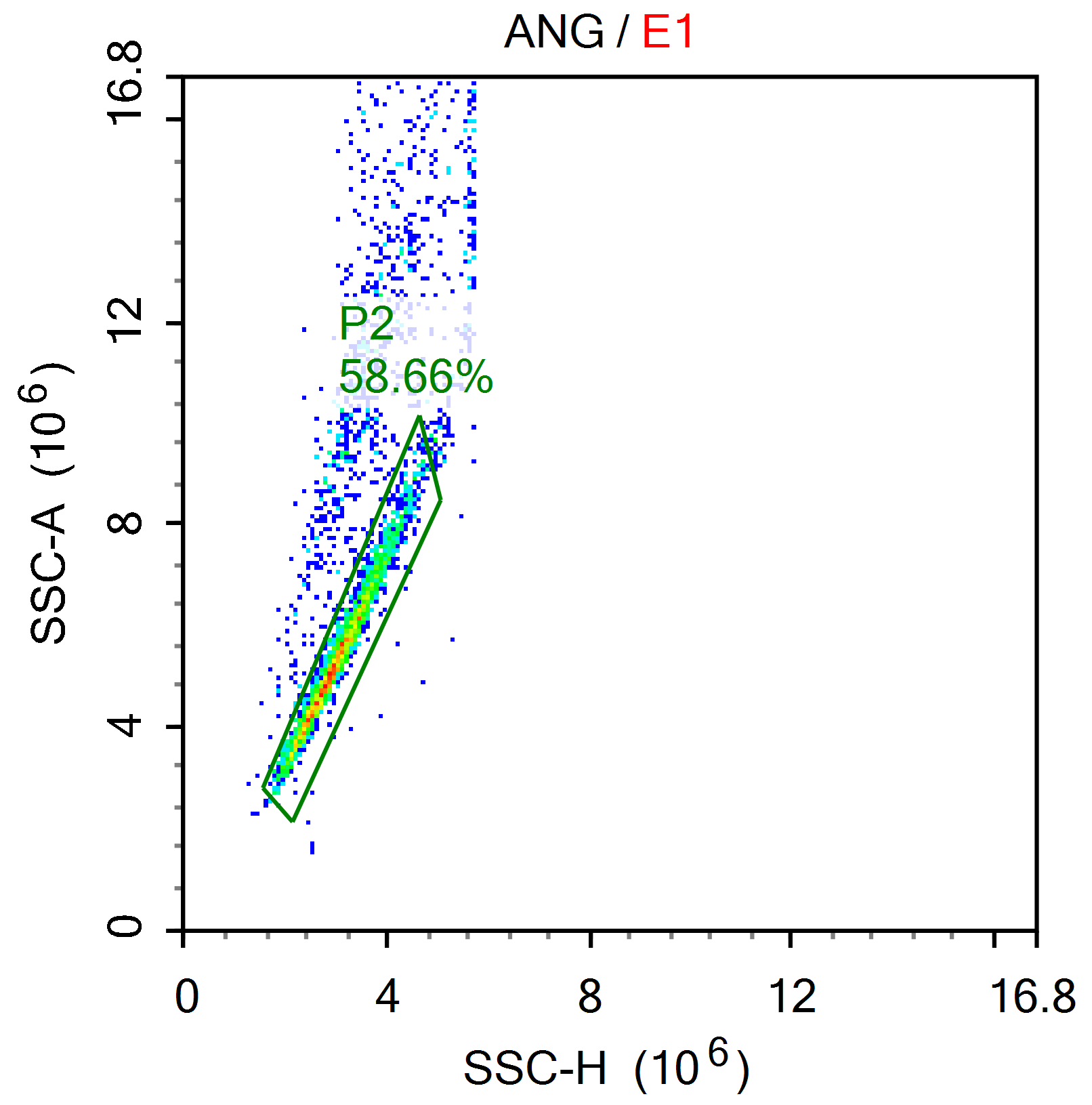

Supplement: Supplementary file 1 [file DataSheet1.zip › supplementary figure1 data/cell cycle/siANG/Remove diploid analysis.tiff]

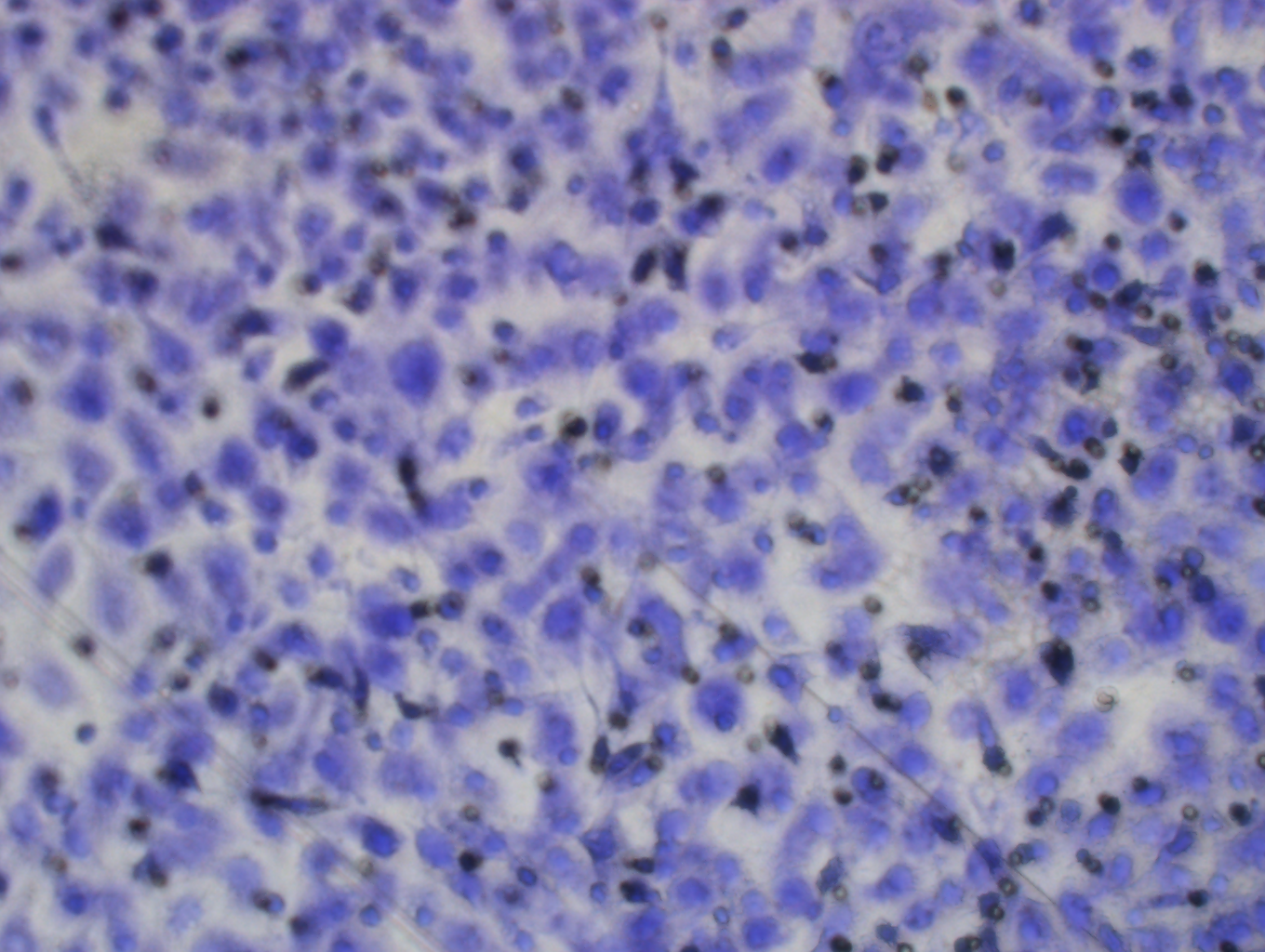

Supplement: Supplementary file 1 [file DataSheet1.zip › supplementary figure1 data/migration-NC.tif]

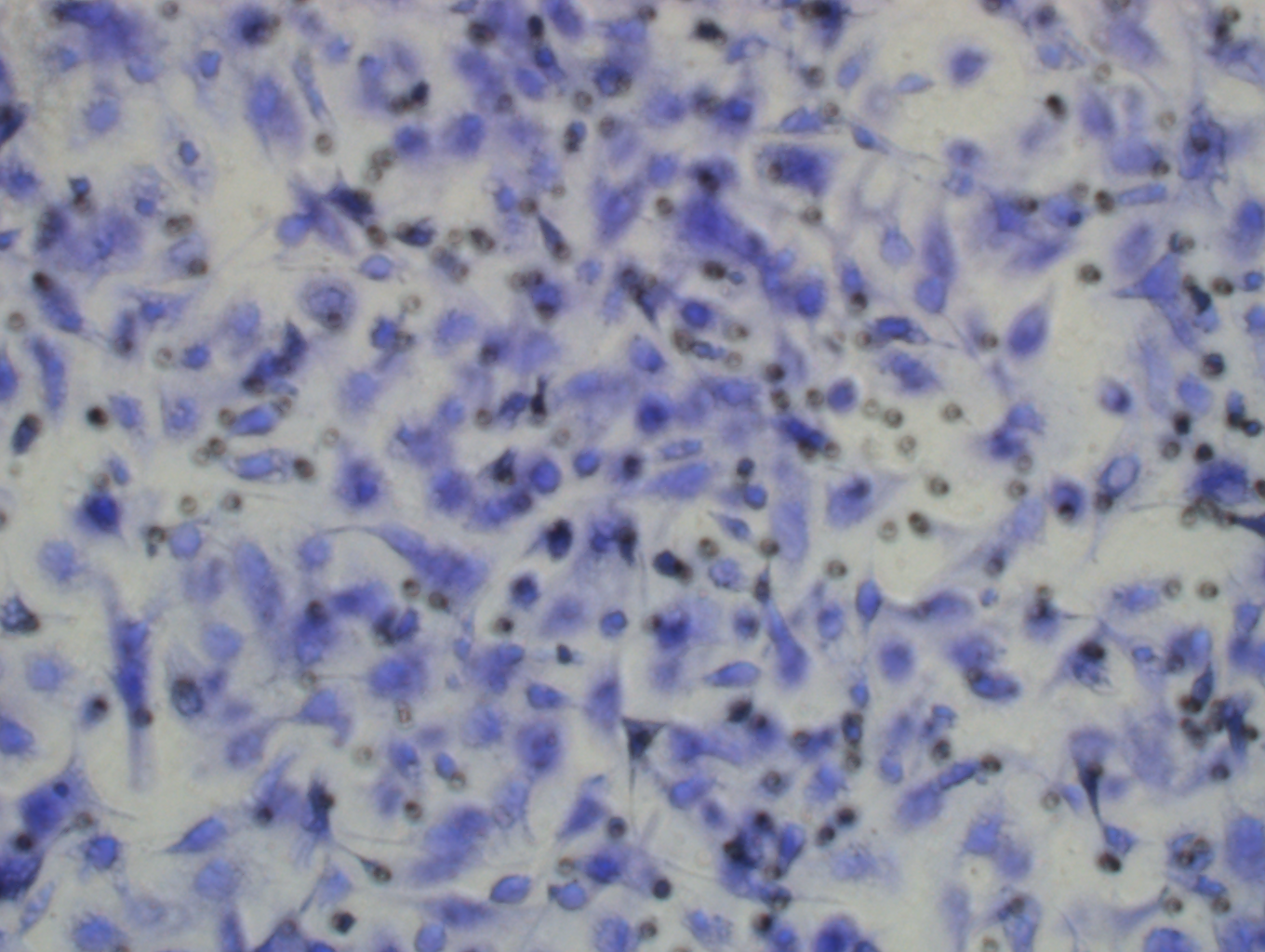

Supplement: Supplementary file 1 [file DataSheet1.zip › supplementary figure1 data/migration-siANG.tif]
